# All‐Inorganic Polyoxometalates Act as Superchaotropic Membrane Carriers

**DOI:** 10.1002/adma.202309219

**Published:** 2023-11-27

**Authors:** Andrea Barba‐Bon, Nadiia I. Gumerova, Elias Tanuhadi, Maryam Ashjari, Yao Chen, Annette Rompel, Werner M. Nau

**Affiliations:** ^1^ School of Science Constructor University Campus Ring 1 28759 Bremen Germany; ^2^ Universität Wien Fakultät für Chemie Institut für Biophysikalische Chemie Josef‐Holaubek‐Platz 2 Wien 1090 Austria

**Keywords:** chaotropic carriers, delivery, Keggin anions, membrane transport, POM stability

## Abstract

Polyoxometalates (POMs) are known antitumoral, antibacterial, antiviral, and anticancer agents and considered as next‐generation metallodrugs. Herein, a new biological functionality in neutral physiological media, where selected mixed‐metal POMs are sufficiently stable and able to affect membrane transport of impermeable, hydrophilic, and cationic peptides (heptaarginine, heptalysine, protamine, and polyarginine) is reported. The uptake is observed in both, model membranes as well as cells, and attributed to the superchaotropic properties of the polyoxoanions. In view of the structural diversity of POMs these findings pave the way toward their biomedical application in drug delivery or for cell‐biological uptake studies with biological effector molecules or staining agents.

## Graphical abstract

Inorganic anions of the polyoxometalate type display a new biological activity: They efficiently transport cationic peptides across lipidic and cellular membranes. This function is related to their superchaotropic nature, resulting in strong and concomitant interactions with peptides and lipids. Mixed‐metal Keggin‐type anions show the required hydrolytic stability and sufficiently low charge density that are prerequisites for the desirable carrier utility.

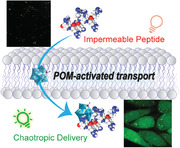

## 1 Introduction

The systematic understanding of the peculiar effects that different ions exert on biological matter can be traced back to Hofmeister, who studied their interactions with proteins.^[^ ^1^, ^2^, ^3^ ^]^ This has led to the formulation of the classical Hofmeister scale, where ions were subsequently classified as being kosmotropic if they showed salting‐out properties toward proteins and as chaotropic if they displayed salting‐in effects.^[^ ^4^, ^5^, ^6^ ^]^ Subsequently, these hydration effects have been generalized, the notion that the effects are a consequence of property changes of the bulk liquid has been abandoned, and, instead, direct interactions between chaotropic ions and dissolved organic matter (“chaotropic effect”) have moved into the focus.^[^ ^7^, ^8^, ^9^ ^]^ The traditional examples of chaotropic anions are SCN^−^ and ClO_4_^−^, with BF_4_^−^ and PF_4_^−^ added later.

Although inorganic cluster anions of the boron and polyoxometalate (POM)^[^ ^10^, ^11^, ^12^, ^13^ ^]^ type have been intensively investigated for many decades, their chaotropicity has moved into the focus of attention only recently, when it was found that these large cluster ions behave as “superchaotropic” ions, that is, they exceed the chaotropic behavior of the hitherto described anions by far.^[^ ^8^, ^14^, ^15^, ^16^, ^17^, ^18^ ^]^ Since the utilization of boron clusters of the dodecaborate (B_12_X_12_^2‒^) and metallacarborane (COSAN) type as membrane carriers has been introduced recently,^[^ ^19^, ^20^ ^]^ and their activity could be traced back to their superchaotropic nature,^[^ ^8^, ^14^, ^21^, ^22^ ^]^ this new application potential needs to be scrutinized for POMs, which offer even larger chemical diversity than boron clusters.

POMs are a large group of discrete polynuclear metal‐oxo‐anions^[^ ^10^, ^23^, ^24^ ^]^ that are typically recognized as being highly hydrophilic and water soluble. The rapidly growing number of biological POM applications^[^ ^25^, ^26^, ^27^, ^28^, ^29^, ^30^, ^31^ ^]^ entails the need for a deeper understanding and analysis of the mechanism of their action. For example, their unspecific interactions with proteins^[^ ^32^, ^33^ ^]^ and lipids^[^ ^34^, ^35^, ^36^ ^]^ have recently been interpreted in terms of the chaotropic effect and their superchaotropic character.^[^ ^15^ ^]^ On the other hand, the omnipresent tendency for speciation and degradation of POMs in aqueous solution, frequently pronounced near neutral pH,^[^ ^11^, ^37^, ^38^ ^]^ hinder their biological and in particular clinical use,^[^ ^29^ ^]^ which until today is largely focused on antimicrobial or toxic effects, including anticancer,^[^ ^28^, ^39^ ^]^ antiviral,^[^ ^40^, ^41^, ^42^ ^]^ antibacterial,^[^ ^27^, ^43^ ^]^ and enzyme inhibitory^[^ ^33^, ^44^, ^45^, ^46^ ^]^ activity. Herein we report the development of POMs which are sufficiently stable near neutral pH to act as membrane carriers. This constitutes an entirely new biological functionality of POMs, with consequences also for the interpretation of their previously documented effects.

## 2 Results and Discussion

**Figure 1:**
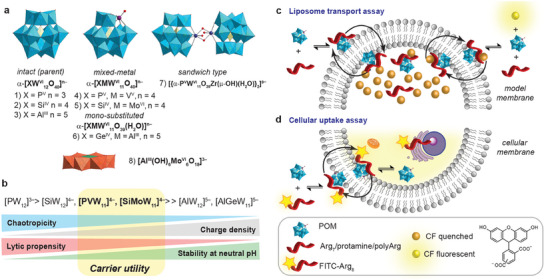
a) POMs evaluated in this study, including the Keggin (top, 1–7) and Anderson (8, bottom) archetypes. (Color code: {WO_6_} blue; {MoO_6_}, orange; {XO_4_} (X = P^V^, Si^IV^, Ge^IV^, Al^III^), yellow; substituting transition ion V^V^, Mo^VI^, purple; Zr^IV^, dark blue; O, red; compounds were used as acid or sodium, potassium, or tetraalkylammonium salts. b) Ordering of the studied POMs according to their chaotropicity (projected based on their charge density), their experimental pH stability, and expected membrane lytic propensity; the window of POMs with practical utility as membrane carriers is shown in yellow. c) Schematic representation of the transport of impermeable oligoarginines facilitated by POMs in model membranes. d) Cellular uptake of the labeled oligoarginine assisted by POMs.

We selected POMs of the globular Keggin and planar Anderson archetype and with varying charge status from −3 to −8 (**Figure** **1**a). To offset the known hydrolysis propensity of [XW_12_O_40_]*^n^* ^‒^ (X = hetero‐ion) in aqueous solution,^[^ ^11^ ^]^ especially at neutral pH, more highly charged mixed‐metal derivatives (Figure 1a, Structures 4–6) were also included. The latter are obtained by hydrolytic removal of one W = O^4+^ unit from the Keggin structure and the subsequent incorporation of an addenda metal to fill the cavity of the monolacunary intermediate.^[^ ^47^, ^48^, ^49^ ^]^ In detail, the following POM anions were studied, see Figure 1a: [PW_12_]^3−^, [SiW_12_]^4−^,^[^ ^50^ ^]^ [PVW_11_]^4−^,^[^ ^48^ ^]^ [SiMoW_11_]^4−^,^[^ ^47^ ^]^ [AlW_12_]^5−^,^[^ ^51^ ^]^ [GeAlW_11_]^5−^,^[^ ^49^ ^]^ [(PZrW_11_)_2_]^8−^,^[^ ^52^ ^]^ and [AlMo_6_]^3−^.^[^ ^53^ ^]^ First, their long‐term (24 h and longer) fate in aqueous solution (slightly acidic and neutral) was investigated by applying the active NMR nuclei in their composition (Table S9 and Figures S24–S37, see the Supporting Information for details). As previously described^[^ ^11^, ^38^ ^]^ the Keggin anions [PW_12_]^3‒^ and [SiW_12_]^4‒^ were found to be fully hydrolyzed at neutral or even slightly acidic pH to their monolacunary forms (Figures S35–S37, Supporting Information), but they were included as negative controls, to exclude potential carrier activity arising from degradation products. The other selected POMs were stable at neutral pH (7.5) or were only slowly hydrolyzed on a long‐time scale, as summarized in **Table** **1**.

**Table 1:** Stability of POMs in acidic and neutral pH and membrane transport parameters of different cargo molecules at pH 7.4

Carrier^a)^	Hydrolysis/stability		HeptaArg^b)^			Protamine^c)^			PolyArg^d)^		
	pH 5.5 ^e)^	pH 7.5 ^f)^	*Y*_max_ ^g)^ [%]	*EC*_50_ ^h)^ [µm]	*E* ^i)^	*Y*_max_ [%]	*EC*_50_ [µm]	*E*	*Y*_max_ [%]	*EC*_50_ [µm]	*E*
[PW_12_]^3‒^	to [P^V^W^VI^_11_O_39_]^7‒^	to [P^V^W^VI^_9_O_34_]^9‒^ ^j)^	‐ – ‐^k)^, ^l)^								
[SiW_12_]^4‒^	to [Si^IV^W^VI^_11_O_39_]^8‒[j]^	to [Si^IV^W^VI^_11_O_39_]^8‒^	‐ – ‐^k)^, ^m)^								
[PVW_11_]^4‒^	Stable	20% to [P^V^W^VI^_11_O_39_]^7‒^	84	49	5.3	88	41	5.9	88	12	8.2
[SiMoW_11_]^4‒^	Stable	55% remain intact	66	17	5.7	72	19	6.0	94	13	8.6
[AlW_12_]^5‒^	/	Stable	‐ – ‐^k)^								
[AlGeW_11_]^5‒^	/	Stable	‐ – ‐^k)^								
[(PZrW_11_)_2_]^8‒^	/	Stable	‐ – ‐^k)^								
[AlMo_6_]^3‒^	/	Stable	‐ – ‐^k)^						33	11	3.1
B_12_Br_12_^2‒^ ^n)^	Stable	Stable	95	48	6.1	84	11	8.0	94	2.6	12
PyBu^‒^	Stable	Stable	100	100	4.8	77	39	5.3	91	12	8.5

^a)^ Carrier activity measured in EYPC vesicles;

^b)^ 10 µm heptaArg;

^c)^ 1 µm protamine;

^d)^ 0.1 µm polyArg;

^e)^ 10 mm 2‐(*N*‐morpholino)ethanesulfonic acid (Mes), 107 mm NaCl, pH 5.5;

^f)^ 10 mm Hepes, 107 mm NaCl, pH 7.4 or 10 mm Tris, 107 mm NaCl, pH 7.4;

^g)^ Maximal activity; ±2% error (SD);

h) Effective concentration to reach 50% of *Y*_max_; 5% error (SD);

^i)^ Activation efficiency *E* = *Y*_max_ (p*EC*_50_/*f*), were p*EC*_50_ is the negative logarithm of *EC*_50_, and *f* a scaling factor set to 20.6 to allocate *E* between 0 and 10;^[^ ^19^, ^55^ ^]^ 10% error (SD, calculated by considering error propagation with respect *Y*_max_ and *EC*_50_);

j) From ref. [11];

^k)^ No detectable activity at pH 5.5 and 7.4;

l) Membrane disruption observed at strongly acidic pH;

^m)^ Carrier activity observed at strongly acidic pH;

^n)^ Measured by HPTS/DPX assay.^[^ ^19^, ^76^ ^]^

**Figure 2:**
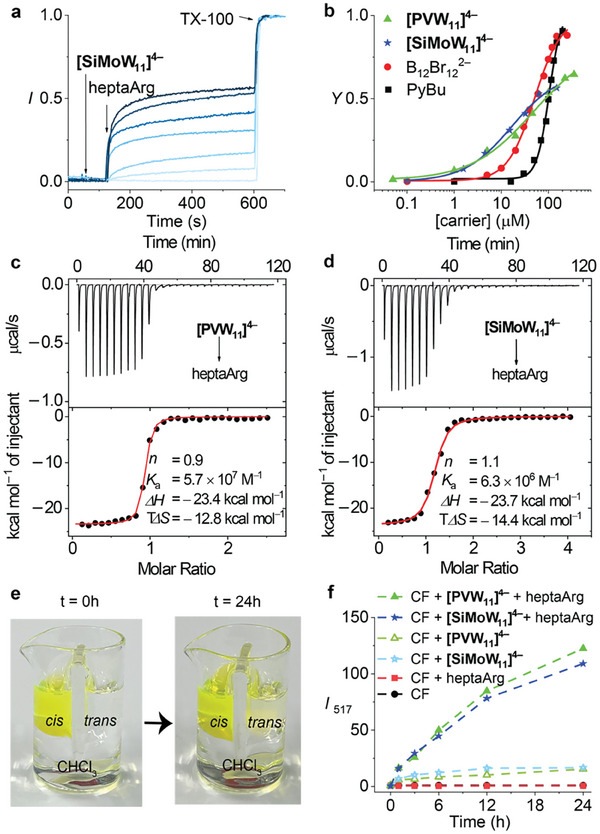
a) Changes in CF emission (*λ*_ex_ = 492 nm, *λ*_em_ = 517 nm) in EYPC⊃CF vesicles as a function of time during the successive addition of i) increasing concentrations of [SiMoW_11_]^4−^ (0–150 µm), ii) heptaArg (10 µm), and iii) TX100, for calibration. b) Dose‐response curves for heptaArg transport for the active POMs and the corresponding Hill curve fits in comparison to the standards. c,d) Microcalorimetric titrations in 10 mm Tris, pH 7.4: thermograms (top) for the sequential injections of c) [PVW_11_]^4−^ or d) [SiMoW_11_]^4−^ into heptaarginine solution, and (bottom) corresponding reaction heats from the integration of the calorimetric traces. POM/oligoarginine concentrations in µm: (c) 100/8 and d) 200/10. e) Pictures of the U‐tube experiment (conducted with [SiMoW_11_]^4−^) at the opening (*t* = 0) and the closing (*t* = 24 h) of the experiment. f) CF fluorescence measured in the *trans* phase of the U‐tube. In the U‐tube experiments, CF, POM, and heptaArg were fixed as 100, 50, and 10 µm.

The capability of POMs to act as membrane carriers was explored first in large unilamellar vesicles (LUVs) loaded with carboxyfluorescein (CF).^[^ ^54^, ^55^ ^]^ We selected three representative cationic peptides, heptaarginine (heptaArg), heptalysine (heptaLys), protamine, and polyarginine (polyArg), as test cargos. Inside the LUVs, the CF dye was encapsulated at self‐quenching concentrations. With a suitable carrier, heptaArg can be taken up into the vesicles, bind with entrapped CF, and shuttle back to the exterior, diluting the previously quenched CF, and thereby restoring its fluorescence (Figure 1c). In the time‐resolved fluorescence experiments (**Figure** **2**a), to test whether a particular POM acts as molecular carrier, the CF emission was continuously monitored during the addition of the inorganic carrier (*t* = 60 s) and after injection of the impermeable selected cargo (*t* = 120 s). Toward the end of the experiment (*t* = 600 s), the surfactant Triton X‐100 was added to release all the entrapped CF and allow normalization of the intensity data.

It should be noted that the addition of POMs alone (without cargo) retained the stability of the liposomes in the studied concentration range (up to 100 µm), that is, neither an increase in CF fluorescence nor changes in size distribution as obtained by dynamic light scattering experiments (DLS, see Figure S4, Supporting Information) were observed, which would have signaled membrane rupture. These negative controls allowed us to proceed to the carrier experiments, with added cargo. Among the eight inorganic clusters evaluated, we found two hits: The two mixed‐metal Keggin POMs **[PVW_11_]^4‒^** and **[SiMoW_11_]^4‒^** triggered the desired increase in fluorescence characteristic for cargo transport. Interestingly, the planar Anderson **[AlMo_6_]^3‒^** also showed incipient transport activity, but only for polyarginine as cargo, while other Anderson‐type POM derivatives were inactive (Figure S9, Supporting Information).

Within the Keggin series, the membrane carrier potential appears to be related to the chaotropicity of the clusters,^[^ ^15^, ^16^, ^18^, ^56^, ^57^ ^]^ which decreases with increasing net charge and charge density, namely **[PW_12_]^3‒^** > **[SiW_12_]^4‒^**, **[PVW_11_]^4‒^**, **[SiMoW_11_]^4‒^** > **[AlW_12_]^5‒^**, **[AlGeW_11_]^5‒^**. When **[PW_12_]^3‒^** and **[SiW_12_]^4‒^** were kept at pH 1.2 (to prevent hydrolysis), already the addition of **[PW_12_]^3‒^** alone to the liposomes caused an increase in fluorescence, showing strong interaction and membrane disruption (Figure S5a, Supporting Information). **[SiW_12_]^4‒^** showed the desired cargo transport at acidic pH, without membrane disruption (Figure S5, Supporting Information). However, above pH 4.5, **[PW_12_]^3‒^** and **[SiW_12_]^4‒^** undergo rapid hydrolysis to afford the highly charged (less chaotropic) products of hydrolysis [P^V^W^VI^_11_O_39_]^7‒^ and [Si^I^ ^V^W^VI^_11_O_39_]^8‒^, such that aged solutions showed neither an interaction with the membrane nor cargo transport (Figures S1–S3, Supporting Information).^[^ ^11^ ^]^ In contrast, the two Al‐based clusters **[AlW_12_]^5‒^** and **[AlGeW_11_]^5–^** are stable at neutral pH (Figures S20–S31, Supporting Information), but their higher charge density appears to adversely affect their transport property. The sandwich cluster **[(PZrW_11_)_2_]^8‒^** was also evaluated (Figure S34, Supporting Information) but neither transport activity nor membrane disruption was observed.

Accordingly, we concentrated on the two mixed‐metal clusters **[PVW_11_]^4‒^** and **[SiMoW_11_]^4‒^**, which showed the desired interplay of sufficiently low net charge (−4), sufficient hydrolytic stability (Figures S28–S30 and S38, Supporting Information) near neutral pH, efficient carrier activity, and broad cargo scope.

To characterize the transport capability of the new inorganic carriers, the resulting normalized fluorescence traces were plotted versus the POM concentration, generating dose‐response curves (Figure 2b; and Figures S1–S3, Supporting Information), which could be analyzed by Hill analysis (see experimental methods, Equation (2)). The resulting membrane transport parameters are the maximal activity (*Y*_max_), the POM concentration that is required to achieve 50% of maximal activity (*EC*_50_), and the activator efficiency^[^ ^55^ ^]^ (*E*), which are compiled in Table 1. For the selected cargos, the two POM carriers **[PVW_11_]^4‒^** and **[SiMoW_11_]^4‒^** show comparable *E* values to the standards in the field, pyrenebutyrate (PyBu), as amphiphilic carrier, and B_12_Br_12_^2‒^, as superchaotropic carrier (Table 1). Interestingly, the transport in the vesicles does not reach the same high final *Y*_max_ levels, which results in a lowering of the *E* values, but the onset for transport activity occurs at much lower carrier concentrations, around 1 µM (Figure 2b).

Accordingly, the required carrier concentrations are desirably low, e.g., the *EC*_50_ value of **[SiMoW_11_]^4‒^** is about a factor of 3 lower (16.5 vs 48 µm) than that of boron clusters, albeit higher than that of metallacarboranes.^[^ ^20^ ^]^ The efficacy of the clusters did not markedly decrease when the sequence of addition was inverted (Figure S6, Supporting Information). This reflects the reversibility of the involved supramolecular interactions, which is not observed, for example, for PyBu, where (irreversible) cluster‐cargo aggregation is known to interfere and significantly reduces its activity (Figure S6, Supporting Information). Another contrast^[^ ^54^, ^58^, ^59^, ^60^ ^]^ to the amphiphilic PyBu is that the carrier activity of the POM carriers was found to be transferable to the less basic heptalysine (Figure S7, Supporting Information); in fact, the activity characteristics for oligoarginine and oligolysine transport were very similar (Table S4, Supporting Information). Note also that the carrier activity of **[PVW_11_]^4‒^** and **[SiMoW_11_]^4‒^** in the model membranes was comparable at pH 5.5 and pH 7.4 for all cargos listed in Table 1 (Table S4, Supporting Information), which showed that the minor hydrolysis observed at high concentration on long time scales at neutral pH (≈20% for **[PVW_11_]^4‒^** and 45% for **[SiMoW_11_]^4‒^** after 24 h) did not affect its carrier activity significantly because the transport experiments were carried out on a much shorter time scale (10 min). It is also important to note that POMs may become stabilized in the presence of lipids^[^ ^61^, ^62^ ^]^ as well as organic matter^[^ ^63^ ^]^ (e.g., peptides), such that the active species may actually be more stable under the actual transport conditions than in the neat buffers used for the long‐term stability studies by NMR and ESI‐MS.

To affect membrane transport, any carrier needs to display an affinity toward the cargo as well as toward the lipid bilayer. Accordingly, the interaction between **[PVW_11_]^4‒^** and **[SiMoW_11_]^4‒^** and the peptides was analyzed by isothermal titration calorimetry (ITC, Figure 2c,d; and Figures S10 and S11, Supporting Information). These experiments showed strong supramolecular interactions (on the order of 10^7^ m ^−1^) with binding governed by the chaotropic effect^[^ ^8^, ^18^, ^56^, ^64^ ^]^ (enthalpically driven, interaction with the peptide backbones, Table S6, Supporting Information) and Coulombic interactions (interaction with arginine and lysine side chains). The strong but reversible intermolecular interactions between the low‐charged POMs and the cationic oligopeptides present an interesting finding, which was measured for both, oligo‐ and polypeptides. Previous ITC studies have focused only on the binding of proteins with highly charged and therefore weakly chaotropic POMs (*n* < −6, Table S3, Supporting Information), where electrostatic interactions were implied as main driving force.^[^ ^65^, ^66^, ^67^, ^68^, ^69^, ^70^, ^71^, ^72^, ^73^ ^]^ Since no nanoaggregates were observed by DLS measurements (see the Experimental Section), the combined data suggest the formation of discrete POM‐peptide complexes of varying stoichiometries.

U‐tube transport experiments (see the Experimental Section, Figure 2e,f) confirmed that **[PVW_11_]^4‒^** and **[SiMoW_11_]^4‒^** act as “real” carriers that can seize and transport a hydrophilic cargo even through a hydrophobic bulk phase (such as CHCl_3_). In the U‐tube experiments, the *trans* phase contained only buffer, and the *cis* phase is loaded with combinations of CF, carrier (POM), and cargo (heptaArg). Aliquots of the *trans* phase are taken and evaluated by fluorescence at different times. The *trans* phase is initially (*t* = 0) nonfluorescent, but the transport of CF from the *cis* phase through the chloroform barrier leads to fluorescence, signaling successful transport. Figure 2f shows that there is no transport in the absence of POM, inefficient transport in the presence of POM alone, and highly efficient transport with both, POM and peptide. This is consistent with translocation of a POM•heptaArg•CF complex through the organic phase, in support of the mechanism in Figure 1c. Accordingly, the superchaotropic properties of POMs cannot only be used for biological membrane transport, but they are transferable to artificial membranes and phase‐transfer processes in general, as recently independently demonstrated for catalysis and dissolution.^[^ ^13^, ^74^, ^75^ ^]^

To better mimic biological membranes, which contain negatively charged components and cholesterol, equivalent transport experiments were performed in anionic liposomes^[^ ^19^, ^20^ ^]^ with the prototypal POM carriers, **[PVW_11_]^4‒^** and **[SiMoW_11_]^4‒^**. These experiments showed that, despite the apparent Coulombic repulsion, both POMs retained their carrier capabilities (Figure S8 and Table S5, Supporting Information), with only slightly reduced net efficiencies. This encouraged us to transfer the experiments to CHO‐K1 (Chinese hamster ovary) cells, after we had confirmed sufficient stability of the most promising POM carriers, **[PVW_11_]^4‒^** and **[SiMoW_11_]^4‒^**, in the cellular nutrient mixture (F‐12 Ham's medium, Figures S26–S28C, Supporting Information).

**Figure 3:**
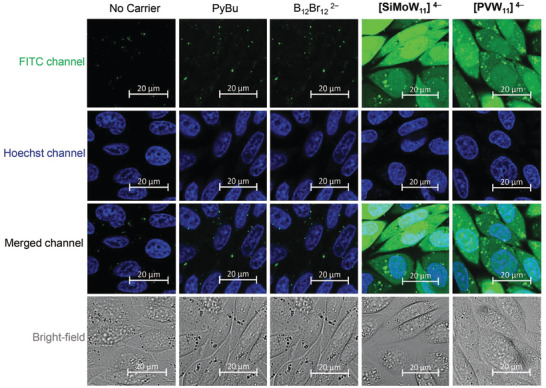
Confocal micrographs of FITC‐Arg_8_ uptake (5 µm) alone (no carrier) and promoted by PyBu, B_12_Br_12_^2‒^, [PVW_11_]^4‒^, and [SiMoW_11_]^4‒^ (5 µm, from left to right) in live CHO‐K1 cells. Representative images of four biological replicates.

POMs are known to cause cellular damage, and their antimicrobial as well as anticancer effects rest on those.^[^ ^28^ ^]^ Nevertheless, we tried to define a “therapeutic window” in which the cytotoxic properties would be sufficiently low to allow their carrier activity to be investigated. Indeed, with up to 10 µm of **[PVW_11_]^4‒^** or **[SiMoW_11_]^4‒^**, CHO‐K1 cells retained about 80% viability after 24 h, according to  resazurin assay (Figure S13, Supporting Information); the onset of carrier activity fell in the same range (Figure 2b; and Figure S8, Supporting Information). Accordingly, the uptake of 5(6)‐fluorescein isothiocyanate‐labelled octaarginine (FITC‐Arg_8_) by these POMs was studied in live CHO‐K1 cells at low carrier concentration (5 µm); the alternative amphiphilic and superchaotropic carriers, PyBu and B_12_Br_12_^2‒^ were used as references (**Figure** **3**). Without carrier, the cells showed minor punctate fluorescence, pointing to (undesirable) endosomal uptake and entrapment of FITC‐Arg_8_.^[^ ^77^, ^78^ ^]^ In the presence of the active stable POMs, however, diffuse fluorescence was observed in both, cytosol and nucleus (Figure 3). Important to note, PyBu and B_12_Br_12_^2‒^ did not achieve efficient uptake at such low carrier concentrations, which is fully consistent with their dose‐response curves obtained from the liposomal model experiments (Figure 2b). Comparable results were obtained in fixed cells (Figure S14–S16, Supporting Information). Colocalization experiments with LysoTracker confirmed cytosolic transport as the major pathway, with only partial endosomal colocalization of FITC‐Arg_8_ (Figure S18, Supporting Information). Accordingly, the two active POMs serve as highly effective intracellular membrane carriers. Interestingly, solutions of the parent Keggin POM, **[PW_12_]^3‒^**, also afforded some apparent cellular uptake, but the cell morphology was highly compromised (Figure S17, Supporting Information), owing to its membrane‐lytic activity (Figure 1b; and Figure S5, Supporting Information).

## 3 Conclusions

Inspired by recent observations of superchaotropic behavior of large cluster ions, we have identified the first two molecular membrane carriers of the all‐inorganic Keggin POM anion type that are functional in both model membranes as well as in live cells and that are stable under physiological conditions. In vesicle and cell experiments, the carriers showed transport activity at concentrations as low as 5 µM that outperform established molecular carriers. They are capable of transporting both arginine‐rich and lysin‐rich oligo‐ and polypeptides. Our experimental findings encourage the optimization of POM carriers, with the aim to further improve their cellular toxicity while maintaining hydrolytic stability. Indeed, we also found an incipient carrier activity for an Anderson‐type POM, which suggests that globular shape presents no rigorous requirement for cluster ions to display this biological functionality. Further chaotropicity and membrane carrier activity modulations could be achieved by incorporation of other hetero‐groups (such as SO_4_^2−^) or by exploring different archetypes such as POMs of the Wells‐Dawson or Lindqvisttype, which is expected to open additional research directions.

## 4 Experimental Section

### Chemicals, Peptides, and Cell Line

Na_3_[α‐P^V^W^VI^_12_O_40_]·nH_2_O ([PW_12_]^3‒^ for [α‐P^V^W^VI^_12_O_40_]^3‒^) was purchased from Sigma‐Aldrich. K_4_[α‐Si^IV^W^VI^_12_O_40_]·14H_2_O ([SiW_12_]^4‒^ for [α‐Si^IV^W^VI^_12_O_40_]^4‒^),^[^ ^50^ ^]^ Na_5_[α‐Al^III^W^VI^_12_O_40_]·12H_2_O ([AlW_12_]^5‒^ for [α‐Al^III^W^VI^_12_O_40_]^5‒^),^[^ ^51^ ^]^ (C_4_H_12_N)_4_[α‐HAl^III^Ge^IV^W^VI^_11_O_39_(H_2_O)]·11H_2_O ([GeAlW_11_]^5‒^ for [α‐Al^III^Ge^IV^W^VI^_11_O_39_(H_2_O)]^5‒^),^[^ ^49^ ^]^ K_4_[α‐P^V^V^V^W^VI^_11_O_40_]·4H_2_O ([PVW_11_]^4‒^ for [α‐P^V^V^V^W^VI^_11_O_40_]^4‒^),^[^ ^48^ ^]^ K_4_[α‐Si^IV^Mo^VI^W^VI^_11_O_40_]·24H_2_O ([SiMoW_11_]^4‒^ for [α‐Si^IV^Mo^VI^W^VI^_11_O_40_]^4‒^),^[^ ^47^ ^]^ (Et_2_NH_2_)_8_[{α‐P^V^W^VI^_11_O_39_Zr^IV^(µ‐OH)(H_2_O)}_2_]·7H_2_O ([(PZrW_11_)_2_]^8‒^ for [{α‐P^V^W^VI^_11_O_39_Zr^IV^(µ‐OH)(H_2_O)}_2_]^8‒^),^[^ ^52^ ^]^ Na_3_[Al^III^(OH)_6_Mo^VI^_6_O_18_]·2H_2_O ([AlMo_6_]^3‒^ for [Al^III^(OH)_6_Mo^VI^_6_O_18_]^3‒^)^[^ ^53^ ^]^ were prepared according to literature procedures. All salts were characterized in solution by NMR spectroscopy and in the solid‐state using infrared (IR) spectroscopy and thermogravimetric analysis (TGA). Trp‐Arg_7_ and Trp‐Lys_7_ were custom‐made by GeneCust (Boynes, France) in >95% purity as confirmed by HPLC and MS by the supplier. Peptide stock solutions were prepared in water and their concentration was determined by using the extinction coefficient of tryptophan at 280 nm (ε = 5540 cm^−1^ m ^−1^).^[^ ^79^ ^]^ FITC‐Arg_8_ was from GL Biochem (Shangai, China) Ltd, and its concentration was determined by using the extinction coefficient of 5‐FITC at 491 nm (*ε* = 73 000 cm^−1^ m ^−1^).^[^ ^80^ ^]^ CHO‐K1 cells were obtained from Sigma‐Aldrich (Steinheim, Germany).

### Vesicle Preparation—Zwitterionic Vesicles in Neutral Media

A thin lipid film was prepared by evaporating a lipid solution of EYPC (egg yolk phosphatidylcholine, 25 mg) in CHCl_3_ (1 mL) with a stream of nitrogen and then dried in vacuo overnight. The dry film was rehydrated with 1 mL buffer (50 mm CF, 10 mm Hepes, pH 7.4 or 50 mm CF, 10 mm Tris, pH 7.4) for 30 min at room temperature, subjected to freeze–thaw cycles (7 times) and extrusions (15 times) through a polycarbonate membrane (pore size 100 nm). Extravesicular components were removed by size exclusion chromatography (NAP‐25 column Sephadex G‐25 DNA grade) with 10 mm Hepes, 107 mm NaCl, pH 7.4, or 10 mm Hepes, 107 mm NaCl, pH 7.4. Phospholipid concentration was calculated by the Stewart assay.^[^ ^81^ ^]^

### Zwitterionic Vesicles in Acidic Media

Acidic vesicles were prepared analogously but the rehydration and elution buffer were 50 mm CF, 10 mm Mes, pH 5.5 and 10 mm Mes, 107 mm NaCl, pH 5.5. respectively.

### Anionic Vesicles in Neutral Media

Anionic vesicles were prepared analogously except for the lipid composition, DMPE/DPPG/CHOL (4.4/10.4/2.6 mg, 1/2/1 molar ratio, DMPE: 1,2‐dimyristoyl‐*sn*‐glycero‐3‐phosphoethanolamine; DPPG: 1,2‐dipalmitoyl‐*sn*‐glycero‐3‐phospho‐(1´‐*rac*‐glycerol); CHOL: cholesterol) in a 1:1 mixture of CHCl_3_ and MeOH (1 mL), the hydration (60 min) and extrusion were performed at 55 °C, and the employed buffers were 100 mm CF, 10 mm Tris, pH 7.4 and 10 mm Tris, 140 mm NaCl, pH 7.4 for rehydration and elution, respectively.

### Transport Experiments

Vesicles stock solutions (5–10 µL) were diluted with the corresponding buffer in a disposable plastic cuvette and gently stirred (total volume 2000 µL, final lipid concentration 13 µm). Carboxyfluorescein fluorescence was monitored at *λ*_em_ = 517 nm (*λ*_ex_ = 492 nm) as a function of time after addition of POMs at 60 s, analyte at 120 s and Triton X‐100 (24 µL 1.2% wt/vol) at 600 s to lyse the vesicles, for calibration. Fluorescence intensities were normalized to fractional emission as

(1) $$I\;\left(t\right)=\frac{I_{t}-I_{0}}{I_{\infty }-I_{0}}\;$$

where *I*_0_ = *I*_t_ before POM addition and *I*_0_ = *I*_∞_ after lysis. For Hill analysis, *I*_t_ before lysis was defined as transport activity, *Y*, and plotted against POM concentration, *c*, and fitted to the Hill Equation (2), to afford the activity in absence of POM, *Y*_0_, the maximal activity, *Y*_max_, the concentration needed to achieve 50% of maximal activity, *EC*_50_, and the Hill coefficient, *n*

(2) $$Y\;=Y_{0}\;+\frac{Y_{\max}-Y_{0}}{1+{\left(\frac{EC_{50}}{c}\right)}^{n}}$$

### Activator Efficiency

In the transport measurements, where different carriers (POMs) are tested with the same cargo, the activator efficiency (*E*) is determined by their capacity to transport the impermeable cargo to the intravesicular region and is characterized by *Y*_max_, its maximal activity, and *EC*_50_, the effective activator concentration. An ideal carrier reaches high *Y*_max_ at low *EC*_50_. To count both factors, the activator efficiency is defined as

(3) $$E\;=Y_{\max}\;\frac{pEC_{50}}{f}$$

were p*EC*_50_ is the negative logarithm of the *EC*_50_, and *f* a scaling factor, which was set to 20.6 to calibrate the highest (previously known) activation efficiency, *E*, as 10.^[^ ^55^ ^]^

### DLS

DLS experiments of the vesicles were carried out on a Malvern Instruments DTS Nano 2000 Zeta‐Sizer. A POM concentration of 100 µm was chosen as default, since this is the highest concentration at which membrane transport was evaluated. The cargo concentration was set to be same as in the transport experiments, namely 10 µm for heptaarginine, 1 µm for protamine, and 0.1 µm for polyarginine. Note that the POMs themselves, with a size of ≈1 nm, fall below the detection limit (>10 nm) of the DLS instrument. Note that DLS measurements of the POMs in the presence of different cargos (at the particular required concentrations) did not afford any detectable signals, which ruled out the formation of nanoaggregates.

### ITC

All experiments were performed with a VP‐ITC MicroCalorimeter from MicroCal, Int,. at atmospheric pressure and 298.15 K. Solutions were degassed and thermostatic prior to the titration experiments in a ThermoVac accessory. A constant volume of POM ([PVW_11_]^4‒^ or [SiMoW_11_]^4‒^) (10 µL per injection) was injected into the oligopeptide solution (heptaArg, protamine, polyArg or heptaLys) in 10 mm Tris, pH 7.4 to determine the apparent binding affinity of [PVW_11_]^4‒^ and [SiMoW_11_]^4‒^ with the oligopeptides. Dilution heats were determined by titration of the POM into buffer and subtracted from the reaction heat. The neat reaction heat was fitted with Origin 7.0 software by using the “one‐set‐of‐sites” model to obtain the complex stability constant (*K*_a_) and molar reaction enthalpy (∆*H*°). The free energy (∆*G*°) and entropy changes (∆*S*°) were obtained according to the relation: ∆*G*° = −R*T*ln*K*_a_ = ∆*H*°−T∆*S*°.

### U‐tube Transport Experiments

The U‐tubes were house‐made, similar to those of Rebek^[^ ^82^ ^]^ and Matile,^[^ ^54^ ^]^ consisting in a small beaker with a central glass barrier, separating the two aqueous layers, *cis* (sampling phase) and *trans* (receiving phase), but allowing an interface chloroform layer below the *cis* and *trans* phases. 3 mL CHCl_3_ are placed into the U‐tube and 1 mL of the *cis* (combinations of carrier (POM), cargo (heptaArg) and CF in buffer) and *trans* (1 mL buffer) phases were added. The organic phase was stirred at 700 rpm at room temperature. Aliquots (25 µL) from the *trans* phase were taken at different times, 400 µL of buffer were added and measured by fluorescence.

### Cell Culture and Confocal Imaging—Fixed Cells

For confocal microscopy experiments, CHO‐K1 cells were seeded into 12‐well plates (Eppendorf) at a density of 1 × 10^5^ cells per well (per 1 mL) and incubated for 24 h in Ham's F‐12 medium containing 10% v/v fetal bovine serum and 1.0% penicillin streptomycin at 37 °C in a 5% CO_2_ atmosphere. Cells were washed twice with PBS, incubated with FITC‐Arg_8_ (4 µm) and carrier (5, 10, and 50 µm) for 1 h at 37 °C, washed twice with Hank´s Balanced Salt solution containing 100 µg mL^−1^ heparin, twice again with PBS, and ultimately fixed with 1 mL cold 4% paraformaldehyde for 20 min. Cells were rewashed twice with PBS and subsequently the nuclei were stained with 2 µg mL^−1^ DAPI for 10 min at ambient temperature before imaging. Cells were imaged by an LSM 980 Airscan2 confocal laser scanning microscope (Zeiss, Germany) and images were processed with the instrument‐specific software ZEN blue 3.7.

### Cell Culture and Confocal Imaging—Living Cells

CHO‐K1 cells were plated into µ‐Slide 8‐well plates (ibidi) at a density of ≈1 × 10^5^ cells per well (per 1 mL) and incubated for 24 h in Ham's F‐12 medium containing 10% v/v fetal bovine serum and 1.0% penicillin streptomycin at 37 °C in a 5% CO_2_ atmosphere. The cells were washed twice with Hank's buffer, incubated for 5 min at 37 °C in Ham's F‐12 medium without phenol red with carrier (5 µm), and subsequently in Ham's F‐12 medium without phenol red with 5 µm FITC‐Arg_8_ for 30 min at 37 °C. Cells in the absence of carrier were used as control. The cells were washed twice with Hank's buffer containing 100 µg mL^−1^ heparin and then washed twice with Ham's F‐12 medium without phenol red. The nuclear staining was performed by incubating the cells with 10 µg mL^−1^ Hoechst 33 342 (bis‐benzimide H33342 trihydrochloride) for 10 min at 37 °C. Cells were directly imaged as stated above.

For colocalization studies live cells were incubated analogously. After medium removal, cells were washed twice with Hank's buffer containing 100 µg mL^−1^ heparin and twice with Ham's F‐12 medium without phenol red. Cellular compartment staining was performed with 10 µg mL^−1^ Hoechst 33 342 (bis‐benzimide H33342 trihydrochloride) and 1 µm LysoTracker Red DND‐99; after 5 min at 37 °C, cells were washed twice with Ham's F‐12 medium without phenol red, and imaged within 5−10 min as described above.

### Cellular Viability Assay

For cell viability assay in thepresence of activators, CHO‐K1 cells were incubated in Ham's F‐12 medium containing 10% fetal bovine serum and 1.0% penicillin streptomycin at 37 °C in a 5% CO_2_ for 24 h. CHO‐K1 cells were dispersed in a 96‐well flat bottom tissue culture treated plates (Eppendorf) at density of 5 × 10^3^ cells per well (per 100 µL) and incubated in Ham's F‐12 containing 10% v/v fetal bovine serum at 37 °C in a 5% CO_2_ incubator for 12 h to allow cell adhesion. Then, cells were incubated with POM, B_12_Br_12_^2−^, or PyBu (10, 50, and 100 µm) for another 24 h. After this incubation time, 20 µL resazurin solution (0.15 mg mL^−1^) was added to cells and incubated for another 3 h. Finally, the plates were read at *λ*_em_ = 590 nm (*λ*_ex_ = 560 nm) using a JASCO fluorometer FP‐8500. The cell viability was evaluated by the following equation

(4) $$\mathrm{Cell}\;\mathrm{viability}=\frac{FI_{\mathrm{sample}}-FI_{\mathrm{blank}}}{FI_{\mathrm{control}}-FI_{\mathrm{blank}}}\times 100\%$$

where, *FI*_sample_ is the fluorescence intensity of cells incubated with different compounds, *FI*_blank_ is the fluorescence intensity of medium, and *FI*_control_ is the fluorescence intensity of cells in absence of any compound.

### Statistical Analysis

Kinetic experiments were performed by triplicates. Data were normalized according to Equation (1) and analyzed using Origin 8.0. Cellular viability was calculated according to Equation (4). Confocal images are representative of four biological replicates. ITCs were performed by duplicates and fitted with Origin 7.0. Data are presented as mean ± standard deviation (SD).

## Conflict of Interest

The University of Vienna has filed a patent application on the use of polyoxometalates for membrane transport.

## Supporting information

Supporting Information

## Data Availability

The data that support the findings of this study are available in the Supporting Information of this article.
