# Supplementary material for: All‐Inorganic Polyoxometalates Act as Superchaotropic Membrane Carriers
Source: Adv Mater. 2023 Nov 27;36(1):2309219. doi: 10.1002/adma.202309219 (PMC11475408; doi:10.1002/adma.202309219)
Supplement: Supplementary file 1 — Supporting Information [file ADMA-36-2309219-s001.pdf]

ADVANCED MATERIALS

Supporting Information

for *Adv. Mater.*, DOI 10.1002/adma.202309219

All-Inorganic Polyoxometalates Act as Superchaotropic Membrane Carriers

Andrea Barba-Bon, Nadiia I. Gumerova, Elias Tanuhadi, Maryam Ashjari, Yao Chen, Annette Rompel and Werner M. Nau**

Supporting Information

All-inorganic polyoxometalates act as superchaotropic membrane carriers

Andrea Barba-Bon, Nadiia I. Gumerova, Elias Tanuhadi, Maryam Ashjari, Yao Chen, Annette Rompel, and Werner M. Nau**

Table of Contents

1. Methods ... 2
2. Literature survey ... 3
3. Transport activity in model membranes ... 5
   3.1. Keggin-type POMs ... 5
      3.1.1. Zwitterionic membranes ... 5
      3.1.2. Anionic membranes ... 8
   3.2. Anderson-type POMs ... 9
4. Supramolecular interactions ... 10
5. Hydrolytic activity of $[(PZrW_{11})_2]^{8-}$ ... 12
6. Transport in cellular membranes ... 13
7. IR spectroscopy ... 16
8. Thermogravimetric analysis ... 19
9. NMR-spectroscopic characterization of identity and stability of the POMs ... 20
   9.1. ^{31}P and ^{51}V NMR spectra of $[PVW_{11}]^{4-}$ ... 22
   9.2. ^{183}W NMR spectra of $[SiMoW_{11}]^{4-}$ ... 27
   9.3. ^{27}Al NMR spectra of $[AlMo_6]^{3-}$ ... 29
   9.4. ^{27}Al NMR spectra of $[AlW_{12}]^{5-}$ ... 30
   9.5. ^{27}Al NMR spectra of $[GeAlW_{11}]^{5-}$ ... 30
   9.6. ^{31}P NMR spectra of $[(ZrPW_{11})_2]^{8-}$ ... 31
   9.7. ^{31}P NMR spectra of $[PW_{12}]^{3-}$ ... 32
   9.8. ^{183}W NMR spectra of $[SiW_{12}]^{4-}$ ... 33
10. ESI-MS ... 34
11. References ... 35

1. Methods

All reagents and chemicals were of high-purity grade and were used as purchased without further purification. Bis-benzimide H 33342 trihydrochloride (Hoechst 33342), carboxyfluorescein (CF), cholesterol, CHO-K1, dulbecco's phosphate buffered saline (DPBS), 1,2-dimyristoyl-sn-glycero-3-phosphoethanolamine (DMPE), 1,2-dipalmitoyl-sn-glycero-3-phospho-(1'-rac-glycerol) (DPPG), egg yolk phosphatidylcholine (EYPC), Hank´s balanced salt solution, nutrient mixture F-12 Ham, lucigenin (LCG), protamine, pyrenebutyrate, *p*-sulfonatocalix[4]arene (CX4) were from Sigma-Aldrich (Steinheim, Germany). Lysotracker™ Red DND-99 was from Molecular Probes (Eugene, Oregon, USA). Resazurin was purchased from TCI (Eschborn, Germany).

Attenuated total reflection Fourier−transform Infrared Spectroscopy
All FTIR spectra were recorded on a Bruker Vertex 70 IR Spectrometer equipped with a single−reflection diamond−ATR unit. Frequencies are given in cm^{-1}, intensities denoted as w = weak, m = medium, s = strong, br = broad.

Elemental analysis
Elemental microanalysis of C/H/N/O contents was performed by Mikroanalytisches Laboratorium (University Vienna, Faculty of Chemistry). An EA 3000 (Eurovector) was used for C/H/N/O-analysis. O-determination was performed by high temperature digestion using the HT 1500 (Hekatech, Germany) pyrolysis system in combination with the EA 3000 system.

Thermogravimetric analysis (TGA)
TGA was performed on a Mettler SDTA851e Thermogravimetric Analyzer under N_2 flow with a heating rate of 5 K min^{-1} in the region 298−873 K.

Fluorescence measurements
Fluorescence measurements and kinetic peptide hydrolysis assays were performed on a Varian Cary Eclipse spectrofluorometer.

Electrospray Ionization Mass Spectrometry (ESI-MS)
Mass spectra were obtained with a timsTOF flex LC-MS System supplied by Bruker Daltonics Ltd. Bruker Daltonics Data Analysis 4.0 software was used to analyze the results.

2. Literature survey

Table S1. Survey of experimentally proven membrane penetration of POMs according to a search in Scopus (“polyoxometalate” and “membrane penetration”) up to the submission date of the manuscript.

POM	Investigated biological activity; pH, buffer	Method to prove membrane penetration	Active POM species; determined by method	Reference
In cells				
$K_{10}[Co_4(H_2O)_2(PW_9O_{34})_2]\cdot nH_2O$ (n is not specified in the article)	Anticancer activity against human T-lymphocyte cell line (MT2 cells) in cell culture medium RPMI (Gibco Roswell Park Memorial Institute) -1640[a)]	Scanning proton microprobe, proton-induced X-ray emission	No stability studies	[1]
$K_{12}H_2[P_2W_{12}O_{48}]\cdot 24H_2O$, $K_{10}[P_2W_{18}Zn_4(H_2O)_2O_{68}]\cdot 20H_2O$ and $[Me_3NH]_8[Si_2W_{18}Nb_6O_{77}]$	Antiviral activity in J774 macrophages in Dulbecco's modified Eagle medium (DMEM) pH 7.4	*In vitro* fluorescence microscopy, SEM, TEM	No stability studies	[2]
Liposome-encapsulated Keggin-type polyoxometalate $K_6[SiW_{11}TiO_{40}]\cdot 16H_2O$	Antitumor activity against KB (human epithelial carcinoma) and HeLa (cervical) cancer cells in phosphate saline buffer (PBS)[b)] at pH 7.4	ICP elemental analysis	In PBS at pH 7.4 $[SiW_{11}TiO_{40}]^{6-}$ UV/Vis, CV, ^{183}W NMR	[3]
Polyoxotungstate lacunary scaffolds, $[\gamma\text{-}SiW_{10}O_{36}]^{8-}$ (SiW_{10}) and $[A\text{-}\alpha\text{-}PW_9O_{34}]^{9-}$ (PW_9), functionalized with fluorescent small molecules (dansyl-, pyrene- and fluorescein): $(nBu_4N)_4[\gamma\text{-}SiW_{10}O_{36}\{((CH_3)_2N)C_{10}H_6SO_2NH(CH_2)_3Si\}_2O]$, $(nBu_4N)_4[\gamma\text{-}SiW_{10}O_{36}\{(C_{16}H_9)SO_2NH(CH_2)_3Si\}_2O]$, $(nBu_4N)_4[\gamma\text{-}SiW_{10}O_{36}\{(C_{20}H_{11}O_5)NHC(S)NH(CH_2)_3Si\}_2O]$,$(nBu_4N)_4[\alpha\text{-}PW_9O_{34}\{(C_{20}H_{11}O_5)NHC(S)NHCH(CH_3)PO\}_2]$	Cell uptake and distribution studies on Human embryonic kidney 293 cells in Dulbecco's Modified Eagle Medium (DMEM)[c)]	*In vitro* fluorescence microscopy	In PBS at pH 7.4 by UV/Vis and DLS up to 48 h showing a minor change of the spectral features, ascribed to aggregation phenomena	[4]
In model membrane				
Keggin-type (Fig. 1A) $SiW_{12}O_{40}^{4-}$, $PW_{12}O_{40}^{3-}$, Dawson-type $P_2W_{18}O_{62}^{6-}$	The interaction between POM and a model cell membrane (egg-phosphatidylcholin) in Tris/HCl buffer (pH 8.5) was investigated.	Leakage experiments	No stability studies	[5]
Keggin-type (Fig. 1A) $H_4[SiW_{12}O_{40}]$	The interaction between POM and a model cell membrane (egg-phosphatidylcholin/Texas Red 1,2-dihexadecanoyl-sn-glycero-3-phosphoethanolamine (TR-DHPE/egg-PC) bilayer) in 150 mM Tris HCl buffer (pH = 7.42) was tested. This study implied that the POM can form and release surfactant-encapsulated cluster, which is accompanied by pore formation in the membrane.	Single-molecule observation by total internal reflection fluorescence microscopy	No stability studies	[6-7]

a) for more detailed information about buffer composition: https://www.thermofisher.com/at/en/home/technical-resources/media-formulation.114.html.
b) PBS contains Na_2HPO_4, KH_2PO_4, NaCl, KCl.
c) for more detailed information about buffer composition: https://www.thermofisher.com/at/en/home/technical-resources/media-formulation.8.html.

Table S2. Biological activity of $\mathbf{[PVW_{11}]^{4-}}$ and $\mathbf{[SiMoW_{11}]^{4-}}$ (or their precursors or decomposition products), the two POMs that have shown membrane transport activity in our present study, up to the submission date of the manuscript.

POM	Activity	Experimental conditions	Active POM species; determined by method	Reference
$K_4[\alpha\text{-}P^VV^VW^{VI}{}_{11}O_{40}]\cdot 2H_2O$ ($\mathbf{[PVW_{11}]^{4-}}$) in combination with β-Lactamase	Low antibacterial effect (MIC = 800 µg/ml, when MIC for oxacillin is 256 µg/ml) against methicillin-resistant *Staphylococcus aureus* (SR3605, ATCC43300)	Mueller-Hinton broth (MHB)[a)] at pH 7.4 and 34 °C	No stability studies	[8]
$[Si^{IV}W^{VI}{}_{11}O_{39}]^{8-}$ (precursor for $\mathbf{[SiMoW_{11}]^{4-}}$) in combination with β-Lactamase	Very low antibacterial effect (MIC = 2000 µg/ml, when MIC for oxacillin is 256 µg/ml) against methicillin-resistant *Staphylococcus aureus* (SR3605, ATCC43300)	Mueller-Hinton broth (MHB)[a)] at pH 7.4 and 34 °C	No stability studies	[9]

a) MHB consists of dehydrated infusion from beef, casein hydrolysate, starch, for more detailed information about buffer composition: https://labmal.com/2019/11/20/mueller-hinton-agar-and-mueller-hinton-broth/

Table S3. Previous reported ITC binding studies of POMs with proteins and/or peptides up to the submission date of the manuscript.

POM	Peptide/Protein	Thermodynamic response	Experimental conditions	Active POM species; determined by method	Reference
Keggin-type $[H_2W_{12}O_{40}]^{6-}$<br>Preyssler-type $[NaP_5W_{30}O_{110}]^{14-}$	Human serum albumin	Enthalpically driven, exothermic process.<br>$[H_2W_{12}O_{40}]^{6-}$: 2.9 x 10^6 M^{-1}(1:1)<br>$[NaP_5W_{30}O_{110}]^{14-}$: not determined.	Tris/HCl 50 mM<br>pH 7.5, 30 ºC	No stability studies.	[10]
		$[H_2W_{12}O_{40}]^{6-}$: 9 x 10^6 M^{-1} (10:1)<br>$[NaP_5W_{30}O_{110}]^{14-}$: 13 x 10^6 M^{-1} (4:1)	pH 3.5		
Wells Dawson<br>α_2-$[P_2W_{17}O_{61}]^{10-}$ and its metal- derivatives:<br>α_2-$[NiP_2W_{17}O_{61}]^{8-}$ and<br>α_2-$[CuP_2W_{17}O_{61}]^{8-}$	Human serum albumin	α_2-$[P_2W_{17}O_{61}]^{10-}$: 3.6 x 10^5 M^{-1} (1:1)<br>α_2-$[NiP_2W_{17}O_{61}]^{8-}$: not determined.<br>α_2-$[CuP_2W_{17}O_{61}]^{10-}$: 2.6 x 10^5 M^{-1} (1:1)	pH 7.5, 30 ºC	No stability studies.	[11]
Sandwich-keggin type<br>$Gd(\beta_2\text{-}Si_2W_{11}O_{39})_2]^{13-}$	Human serum albumin	Enthalpically driven, exothermic process.<br>3.3 x 10^6 M^{-1} (1:1)	Tris/HCl 50 mM<br>pH 7.5, 30 ºC	No stability studies.	[12]
$[EuW_{10}O_{36}]^{9-}$	Human serum albumin	Enthalpically driven, exothermic process.<br>4.2 x 10^6 M^{-1} (1:1)	Tris/HCl 50 mM<br>pH 7.5, 30 ºC	No stability studies.	[13]
Keggin-type $[H_2W_{12}O_{40}]^{6-}$<br>Preyssler-type<br>$[NaP_5W_{30}O_{110}]^{14-}$	Aβ1-40 (amyloid peptide 39-42 amino acids)	Enthalpically driven, exothermic process.<br>$[H_2W_{12}O_{40}]^{6-}$: 2.1 x 10^5 M^{-1}<br>1.3 x 10^5 M^{-1}<br>2.1 x 10^4 M^{-1}<br>$[NaP_5W_{30}O_{110}]^{14-}$: 1.2 x 10^5 M^{-1} (1:1)	30 ºC	No stability studies.	[14]
$[Ce(PW_{11}O_{39})_2]^{10-}$	HEWL (hen egg white lysozyme)	Enthalpically driven, exothermic process.<br>4.9 x 10^6 (1:1)	Phosphate buffer, 10 mM<br>pH 7.4, 25 ºC	No stability studies.	[15]
		1.7 x 10^6 (1:1)	37 ºC		
$[Zr(\alpha_2\text{-}P_2W_{17}O_{61})]^{16-}$	Human serum albumin	Enthalpically driven, exothermic process.<br>2 x 10^8 M^{-1} (1:1)<br>7 x 10^5 M^{-1} (3:1)	Phosphate buffer, 10 mM<br>pH 7.5, 25 ºC	No stability studies.	[16]
$[Eu(SiW_{10}MoO_{39})_2]^{13-}$	Peptide HPV16Ctb	Enthalpically driven, exothermic process.<br>2.4 x 10^9 M^{-1} (1:2) (sept I)<br>1.5 x 10^8 M^{-1} (3:1) (sept II)	Mes-NaOH, 10 mM<br>pH 6,<br>5 ºC	No stability studies.	[17]
		3.2 x 10^9 M^{-1} (1:2) (sept I)<br>1.1 x 10^8 M^{-1} (3:1) (sept II)	10 ºC		
		3.6 x 10^9 M^{-1} (1:2) (sept I)<br>1.0 x 10^8 M^{-1} (3:1) (sept II)	25 ºC		
$[EuW_{10}O_{36}]^{9-}$	HPV16Ctb	Enthalpically driven, exothermic process<br>5.1 x 10^5 M^{-1} (1:1)	Mes-NaOH, 10 mM<br>pH 6, 25 ºC	No stability studies.	[18]
	HPV18Ctb	Enthalpically driven, exothermic process<br>2.3 x 10^6 M^{-1} (1:1)			
	HPV44Ctb	Enthalpically driven, exothermic process<br>3.5 x 10^5 M^{-1} (1:1)			
	HPV5Ctb	Enthalpically driven, exothermic process<br>6.6 x 10^6 M^{-1} (1:1)			
$[EuW_{10}O_{36}]^{9-}$	HPV16 E6 protein	Enthalpically driven, exothermic process<br>6.6 x 10^4 M^{-1} (1:1)	Mes-NaOH, 10 mM<br>pH 6, 25 ºC	No stability studies.	[19]
	HPV16 E6 peptide	Enthalpically driven, exothermic process<br>1.7 x 10^6 M^{-1} (1:1)			
$[Ru_4(\mu\text{-}OH)_2(\mu\text{-}O)_4(\mu\text{-}H_2O)_4(\gamma\text{-}P_2W_{10}O_{36})_2]^{10-}$	CK2 (Ser/Thr protein kinase)	Enthalpically driven, exothermic process<br>2.1 x 10^6 M^{-1} (1:1)	25 mM Tris, 500 mM NaCl<br>pH 8.5, 25.3 ºC	No stability studies.	[20]

3. Transport activity in model membranes

3.1. Keggin-type POMs

3.1.1. Zwitterionic membranes

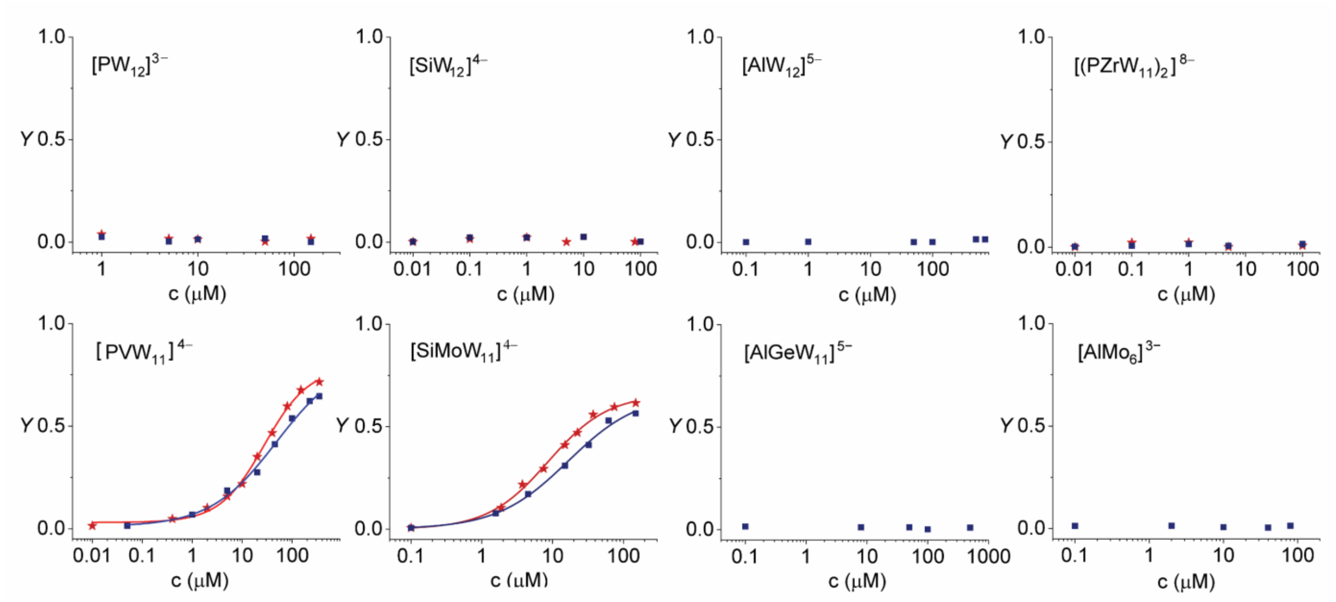

Figure S1. Transport activity, *Y*, for heptaarginine in (10 μM) EYPC⊃CF liposomes as a function of the concentration of the different evaluated POMs. Solid lines correspond to the resulting fit with the Hill equation. Blue squares are data obtained at neutral pH, and red stars data obtained at acidic pH.

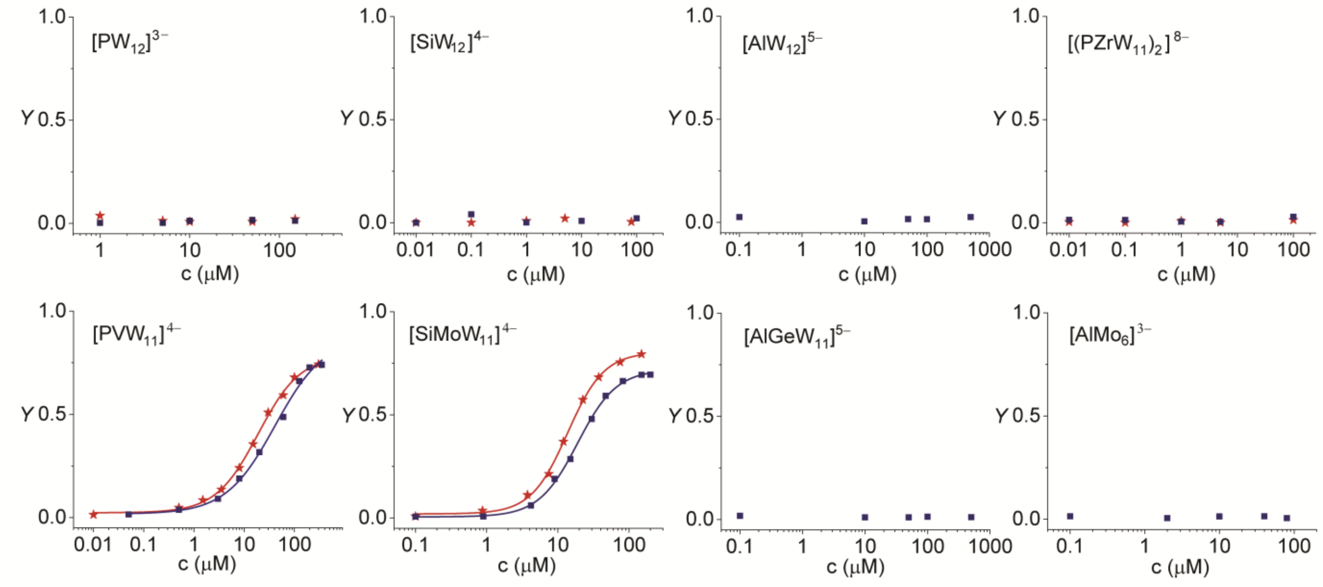

Figure S2. Transport activity, *Y*, for protamine (1 μM) in EYPC⊃CF liposomes as a function of the concentration of the different evaluated POMs. Solid lines correspond to the resulting fit with the Hill equation. Blue squares are data obtained at neutral pH, and red stars data obtained at acidic pH.

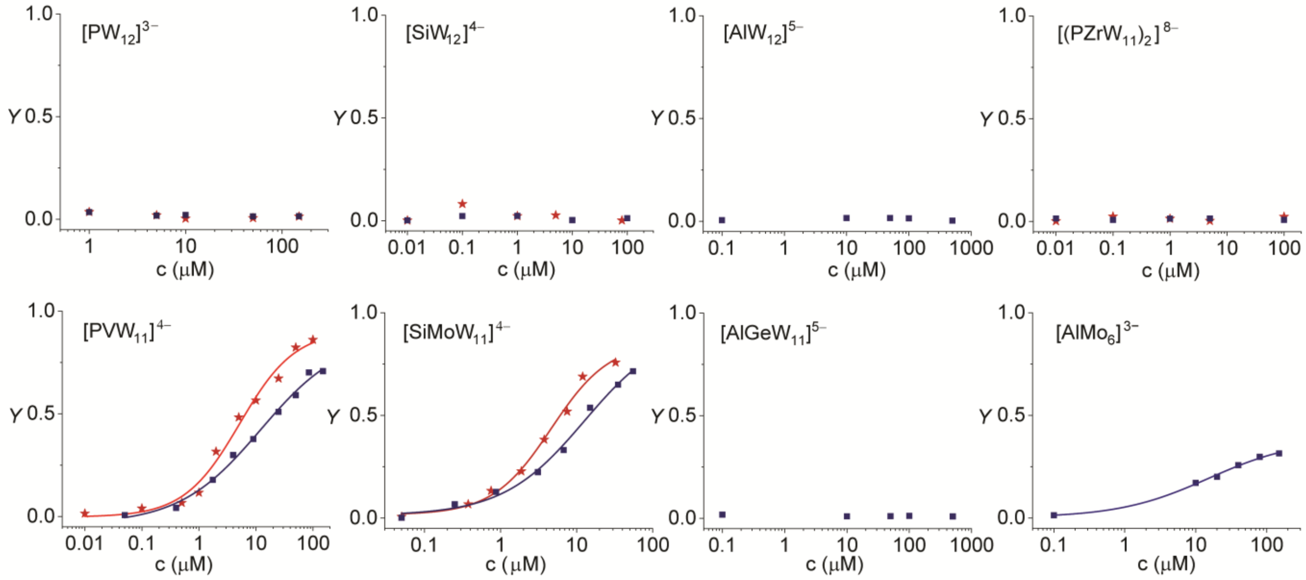

Figure S3. Transport activity, Y, for polyarginine (0.1 μM) in EYPC⊃CF liposomes as a function of the concentration of the different evaluated POMs. Solid lines correspond to the resulting fit with the Hill equation. Blue squares are data obtained at neutral pH, and red stars data obtained at acidic pH.

Table S4. Membrane transport parameters[a)] of active POMs at neutral[b)] and acidic[c)] pH.

carrier	pH	Hydrolysis/Stability	HeptaArg[d)]			Protamine[e)]			PolyArg[f)]		
			Y_{max} [%][g)]	EC_{50}[h)] [μM]	E [i)]	Y_{max} [%]	EC_{50} [μM]	E	Y_{max} [%]	EC_{50} [μM]	E
**[PVW11]4–**	7.5	20% hydrolyzed to $[P^VW^{VI}{}_{11}O_{39}]^{7-}$	8	49	5.3	88	41	5.9	88	12	8.2
	5.5	stable	79	28	5.9	79	19	6.6	91	5	10.1
**[SiMoW11]4–**	7.5	45%	66	17	5.7	72	19	6.0	94	13	8.6
	5.5	stable	66	9	6.6	80	14	7.3	86	5	9.7

a) EYPC vesicles. b) 10 mM Tris, 107 mM NaCl, pH 7.4. c) 10 mM Mes, 107 mM NaCl, pH 5.5. d) 10 μM heptaArg. e) 1 μM Protamine. f) 0.1 μM polyArg. g) Maximal activity; ±2 % error (SD). h) Effective concentration to reach 50% activity of Y_{max}; 5 % error (SD). i) Activation efficiency $E = Y_{max}$ (pEC_{50}/f), were pEC_{50} is the negative logarithm of the EC_{50}, and f a scaling factor set to 20.6 to allocate E between 0 and 10[21], 10% error.

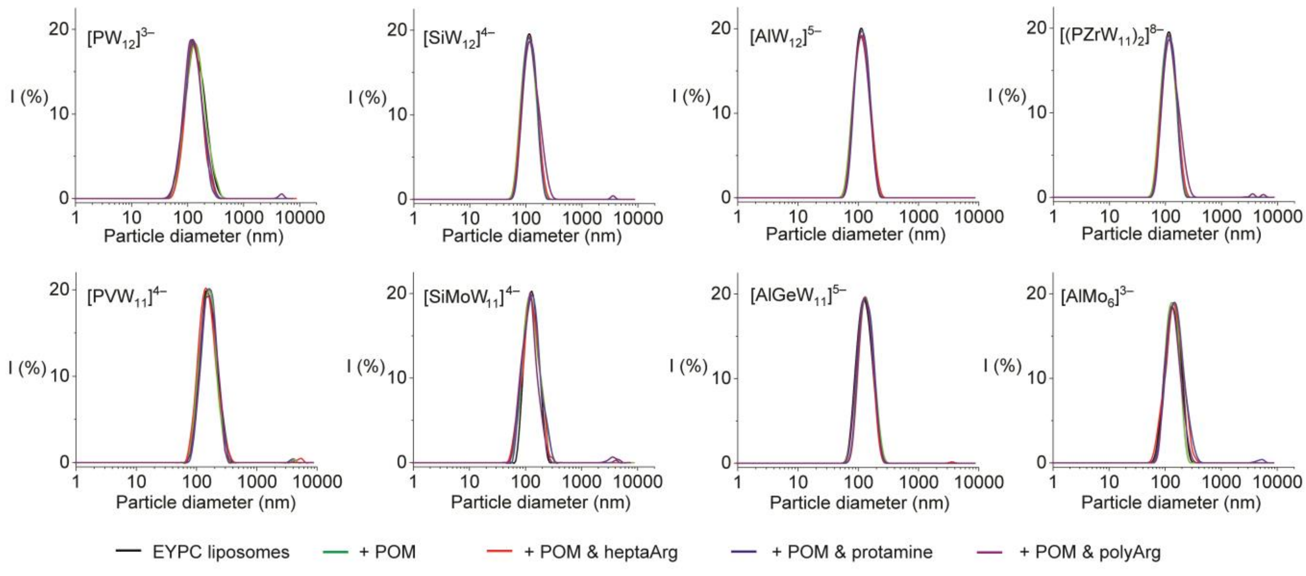

Figure S4. Size distribution (in %, by DLS) of EYPC⊃CF liposomes before (black trace), and after addition of POM alone (100 μM, green trace), and in presence of heptaarginine (10 μM, red trace), protamine (1 μM, blue trace), or polyarginine (0.1 μM, purple trace). Higher concentrations of **[PW12]3–** and **[SiW12]4–** led to vesicle lysis. Experiments were done in 10 mM Tris, 107 mM NaCl, pH 7.4.

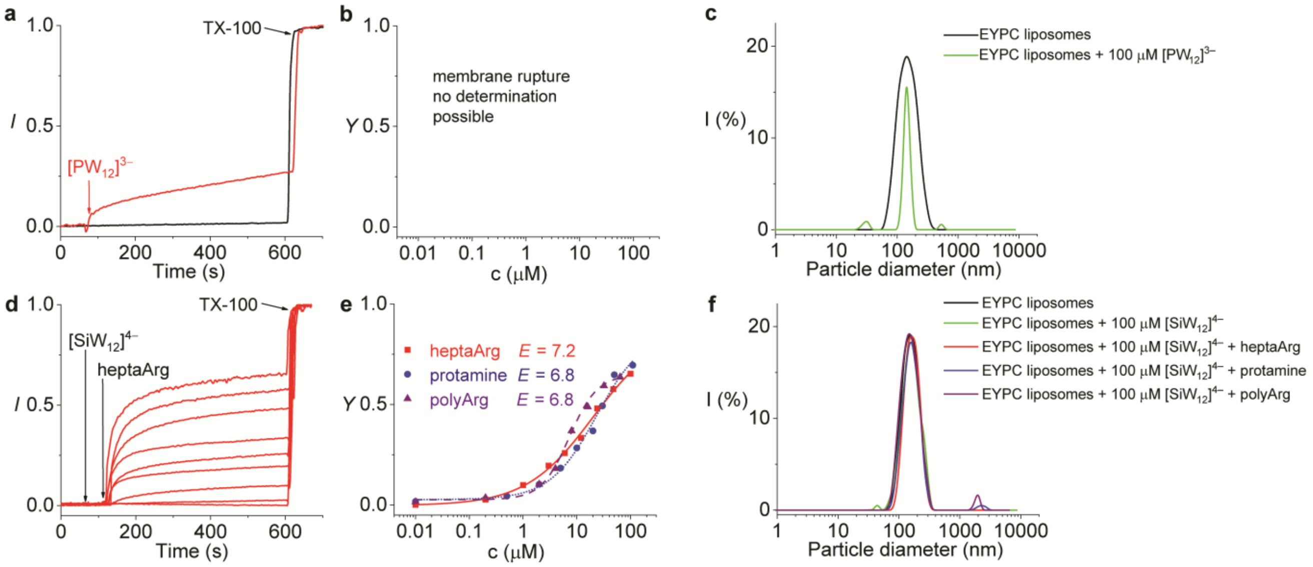

Figure S5. Transport kinetics (left pannels), dose response curves (middle pannels) for cargo transport, and size distribution (by DLS, right pannels) for **[PW12]**$^{3-}$ (top) and **[SiW12]**$^{4-}$ (bottom), measured with EYPC⊃CF liposomes at pH 7.4, but preserving the POM solution at pH 1.2 before addition. As shown in panel **a**, the addition of **[PW12]**$^{3-}$ alone gave a fluorescence increase, pointing to membrane disruption, which was confirmed by DLS (panel **c**). When using **[SiW12]**$^{4-}$ (panel **d**) the resulting pH of the experiment slightly decreased (pH ~ 7.0, for the highest concentrations evaluated); however, this decrease did not affect the membrane stability nor the transport (panels **e** and **f**).

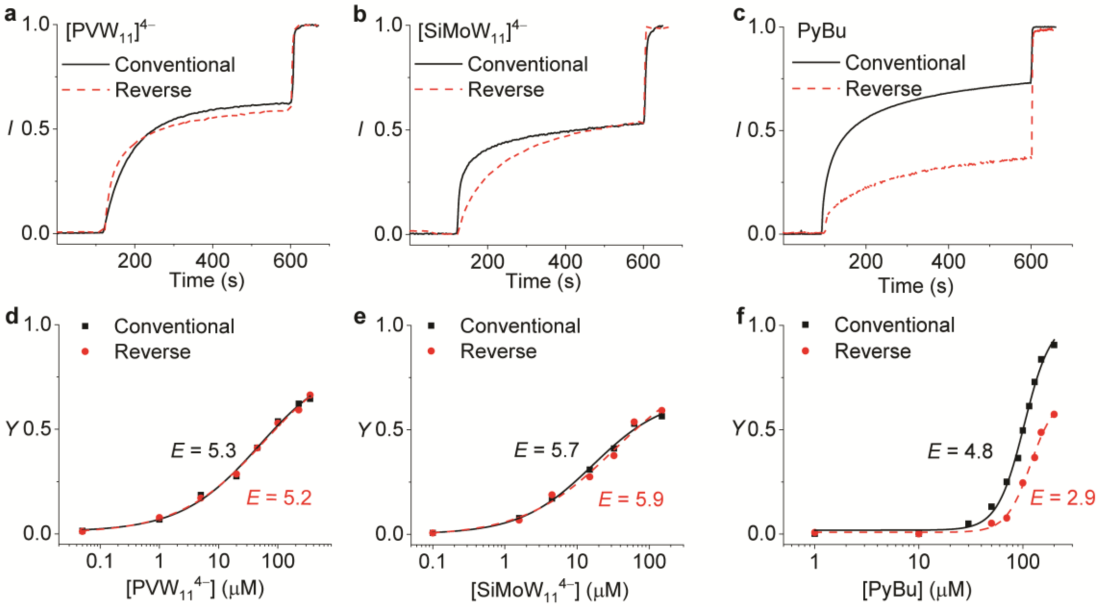

Figure S6. Transport kinetics (upper pannels) and dose response curves (bottom pannels) for the transport of heptaarginine (10 μM) mediated by the superchaotropic POM carriers, **[PVW11]**$^{4-}$ and **[SiMoW11]**$^{4-}$, *vs* PyBu in dependence of carrier-cargo sequence of addition. **a**, 200 μM **[PVW11]**$^{4-}$. **b**, 60 μM **[SiMoW11]**$^{4-}$. **c**, 120 μM PyBu.

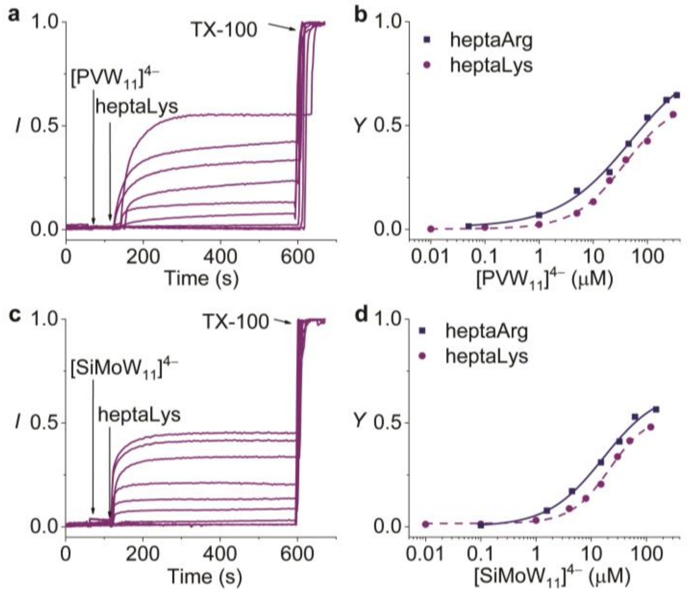

Figure S7. Successful transport of the less basic lysine derivative with **$[PVW_{11}]^{4-}$** (**a**) and **$[SiMoW_{11}]^{4-}$** (**c**). Comparable transport of heptalysine (circles, dash purple line) and heptaarginine (10μM) (squares, solid blue line) activated by **$[PVW_{11}]^{4-}$** (**b**) and **$[SiMoW_{11}]^{4-}$** (**d**).

3.1.2. Anionic membranes

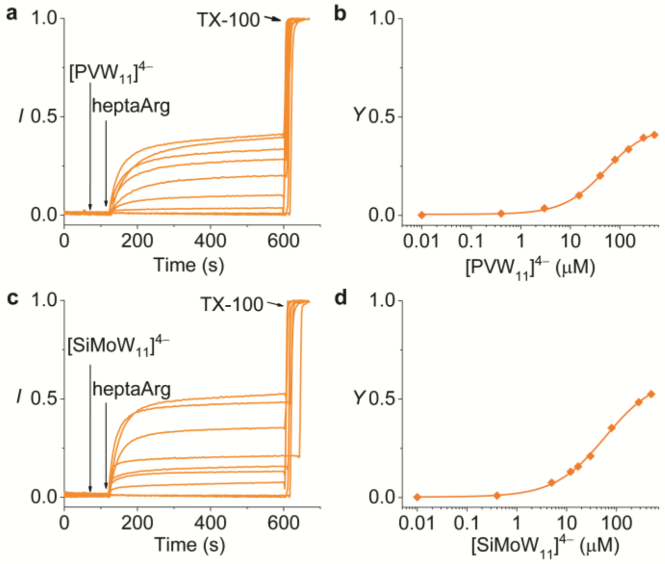

Figure S8. Heptaarginine transport through anionic vesicles[22-23][24] (DMPE/DPPG/CHOL, 1/2/1 molar ratio) facilitated by **$[PVW_{11}]^{4-}$** (**a**) and **$[SiMoW_{11}]^{4-}$** (**c**). Transport activity as function of **$[PVW_{11}]^{4-}$** (**b**) and **$[SiMoW_{11}]^{4-}$** (**d**) concentration, solid lines correspond to the resulting fit with the Hill equation.

Table S5. Membrane transport parameters of active POMs towards different analytes and different lipid composition.

Lipid a)	POM	Cargo b)	Y_{max} [%] c)	EC_{50} d) [μM]	E e)
EYPC *(zwitterionic)*	**$[PVW_{11}]^{4-}$**	heptaArg	84	49	5.3
		heptaLys	62	38	4.3
	**$[SiMoW_{11}]^{4-}$**	heptaArg	66	17	5.7
		heptaLys	54	22	4.4
DMPE/DPPG/CHOL *(anionic)*	**$[PVW_{11}]^{4-}$**	heptaArg	45	50	2.8
	**$[SiMoW_{11}]^{4-}$**	heptaArg	61	60	3.6

a) 13 μM phospholipids. b) 10 μM cargo. c) Maximal activity; ±2% error (SD). d) Effective concentration to reach 50% activity of Y_{max}; 5 % error (SD). e) Activation efficiency $E = Y_{max}$ (pEC_{50}/f), were pEC_{50} is the negative logarithm of the EC_{50}, and f a scaling factor set to 20.6 to allocate E between 0 and 10;[21] 10% error.

3.2. Anderson-type POMs

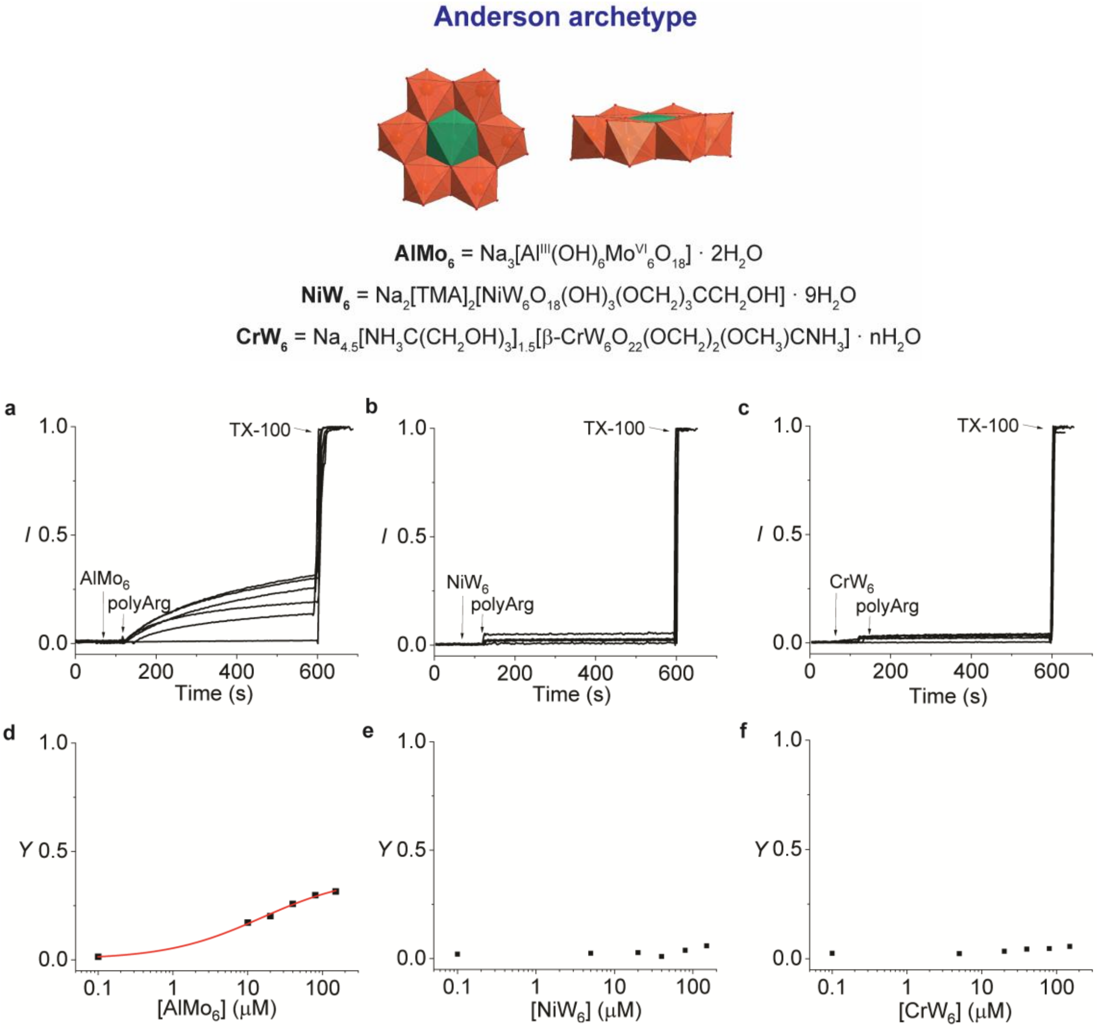

Figure S9.Transport kinetics (upper pannels) and dose response curves (bottom pannels) for polyarginine transport in the presence of **$[AlMo_6]^{3-}$**, **$[NiW_6]^{4-}$**, and **$[CrW_6]^{6-}$**, measured with EYPC⊃CF liposomes at pH 7.4. Note that only **$[AlMo_6]^{3-}$** shows an onset of carrier activity. TMA = tetramethylammonium.

4. Supramolecular interactions

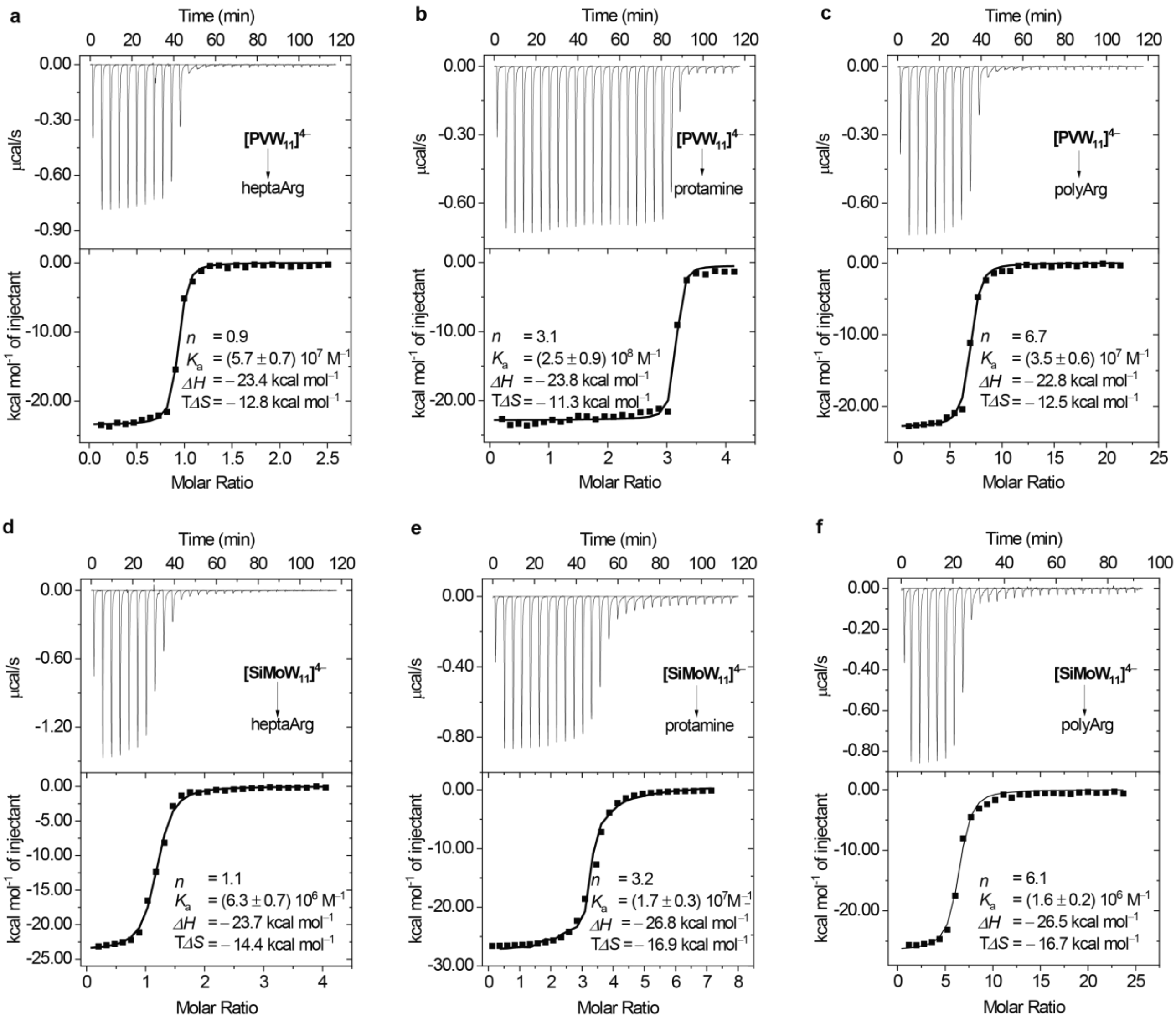

Figure S10. Microcalorimetric titrations in 10 mM Tris, pH 7.4: Raw ITC data (top) for the sequential injections of POM solution into an oligoarginine solution, and apparent reaction heats (bottom) obtained from the integration of the calorimetric traces. POM/oligoarginine concentrations in μM: **a**, 100/8; **b**, 100/5; **c**, 100/1; **d**, 200/10; **e**, 100/3; and **f**, 100/0.9. Error data is 5% for the *n* values and ± 0.5 kcal mol^{-1} for ΔH and $T\Delta S$ (duplicate measurements). Note that none of these interactions lead to any precipitation or aggregates, confirmed by the absence of any detectable signal by DLS.

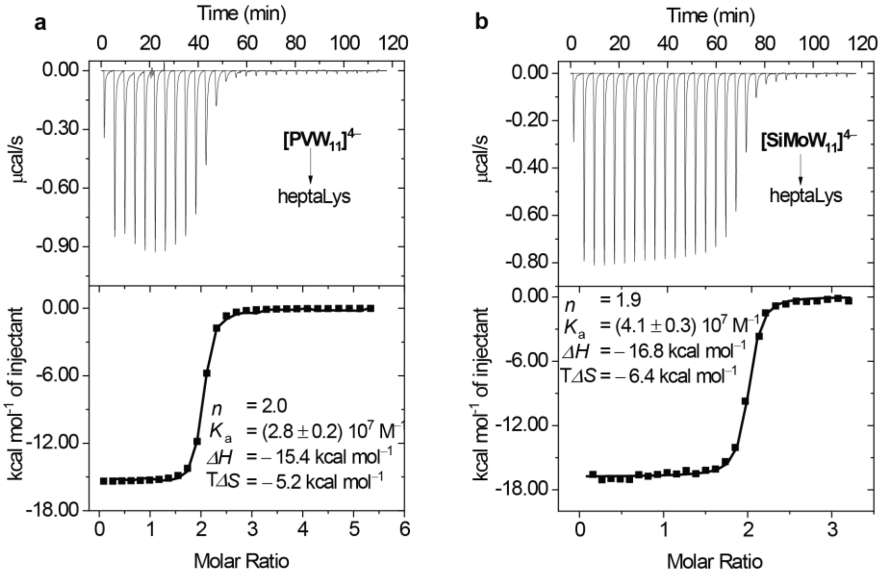

Figure S11. Microcalorimetric titrations in 10 mM Tris, pH 7.4: Raw ITC data (top) for the sequential injections of POM solution into a heptalysine solution, and apparent reaction heats (bottom) obtained from the integration of the calorimetric traces. POM/heptalysine concentrations in μM: **a**, 200/8 and **b**, 150/10. Error data is 5% for the *n* values and ± 0.5 kcal mol^{-1} for ΔH and $T\Delta S$ (duplicate measurements). Note that these interactions did not lead to any precipitation or aggregates, confirmed by the absence of any detectable signal by DLS.

Table S6. Binding constants (K_a in M^{-1}) and thermodinamic data (in kcal mol^{-1}) measured for the complex formation between the stable and active POMs and oligoarginines in 10 mM Tris, pH 7.4.[a)]

	$[PVW_{11}]^{4-}$				$[SiMoW_{11}]^{4-}$			
	n	K_a/ 10^7	ΔH	$T\Delta S$	n	K_a/ 10^7	ΔH	$T\Delta S$
Heptaarginine	0.90	5.7 ± 0.7	–23.4	–12.8	1.1	0.6 ± 0.1	–23.7	–14.4
Protamine	3.1	25 ± 9	–23.8	–11.3	3.2	1.7 ± 0.3	–26.8	–16.9
Polyarginine	6.7	3.5 ± 0.6	–22.8	–12.5	6.1	1.6 ± 0.2	–26.5	–16.7
Heptalysine	2.0	2.8 ± 0.2	–15.4	–5.2	1.9	4.1 ± 0.3	–16.8	–0.4

[a)] Error data is 5% for the *n* values and ± 0.5 kcal mol^{-1} for ΔH and $T\Delta S$ (duplicate measurements).

5. Hydrolytic activity of $[(PZrW_{11})_2]^{8-}$

We devised a supramolecular tandem enzyme assay[25-29] to check to which degree $[(PZrW_{11})_2]^{8-}$ promotes peptide fragmentation, which has been reported to occur on a time scale of days at 60 ºC.[30-31] Supramolecular tandem assay is a technique that allows real-time continuous monitoring of enzymatic activity by following a change in the concentration of a substrate or product as it competitively displaces a fluorescent reporter dye from the cavity of a macrocyclic host. The assay therefore relies on the differential binding of the macrocycle with the fluorescent dye, the enzymatic substrate, and the corresponding product.

We selected *p*-sulfonatocalix[4]arene (CX4) as macrocyclic host and lucigenin (LCG) as fluorescent dye, since protamine (substrate) has a high affinity ($K_a = 1.1 \times 10^9\ M^{-1}$) towards CX4, but shorter peptide fragments (the products resulting from enzymatic fragmentation), show lower affinity (e.g., arginine, $K_a = 2.8 \times 10^3\ M^{-1}$), such that the enzymatic conversion can be directly monitored through a decrease in fluorescence. Trypsin, which is an endopeptidase that hydrolyzes peptide bonds on the C-terminal side of the amino acid arginine,[32-33] was used as an example, for comparison.

Only minor hydrolysis (<10%) of protamine by the $[(PZrW_{11})_2]^{8-}$ was observed on the time scale relevant for the transport experiments (10 min).

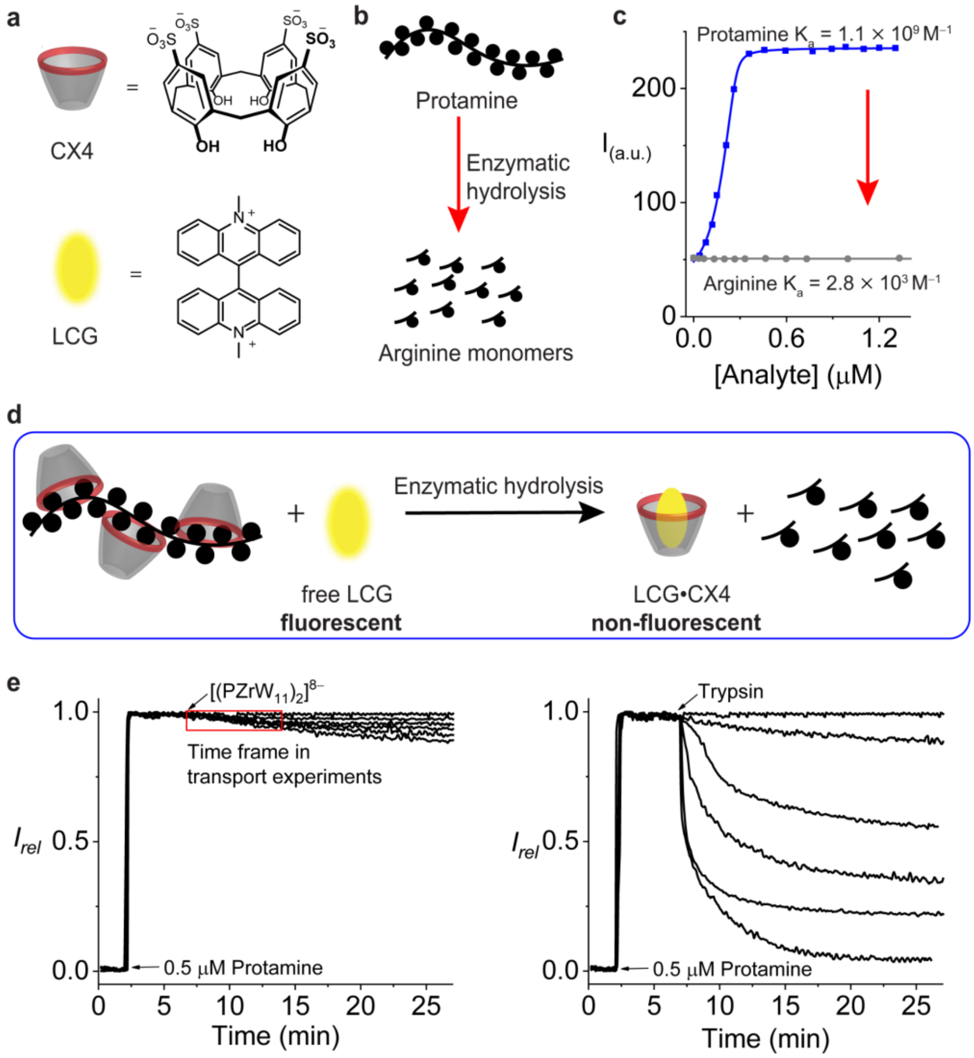

Figure S12: **a** Chemical structure of the macrocyclic host (*p*-sulfonatocalix[4]arene, CX4) and the fluorescent dye (lucigenin, LCG) used as reporter pair in the devised supramolecular tandem enzyme assay. **b** Schematic representation of protamine (as substrate) and its hydrolysis products after enzymatic reaction. **c** Corresponding titration of protamine and arginine by competitive displacement of LCG (0.5 µM) from CX4 (1 µM) in 10 mM Hepes, pH 7.5. **d** Schematic representation of the supramolecular tandem enzyme assay. **e** Time-resolved fluorescence traces of the protamine hydrolysis (λ_{ex} = 369 nm; λ_{em} = 502 nm); 0.5 µM protamine is added to a solution of 0.5 µM LCG and 1 µM CX4, and 5 mins later, increasing concentrations of $[(PZrW_{11})_2]^{8-}$ (0-500 µM, left panel), or trypsin (0-100 nM, right panel).

6. Transport in cellular membranes

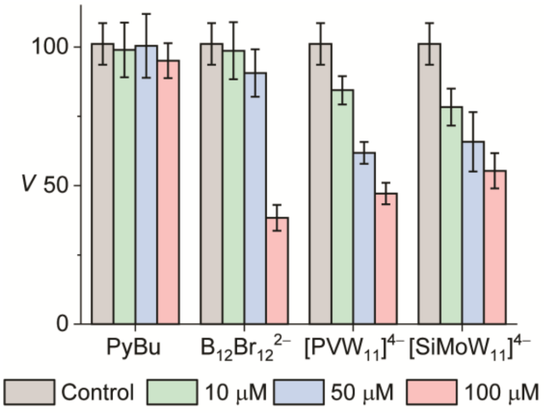

Figure S13. Viability of CHO-K1cells (resazurin assay) incubated with different carrier concentrations (0, 10, 50, and 100 μM) for 24 hours. Error bars refer to standard deviation (n = 3).

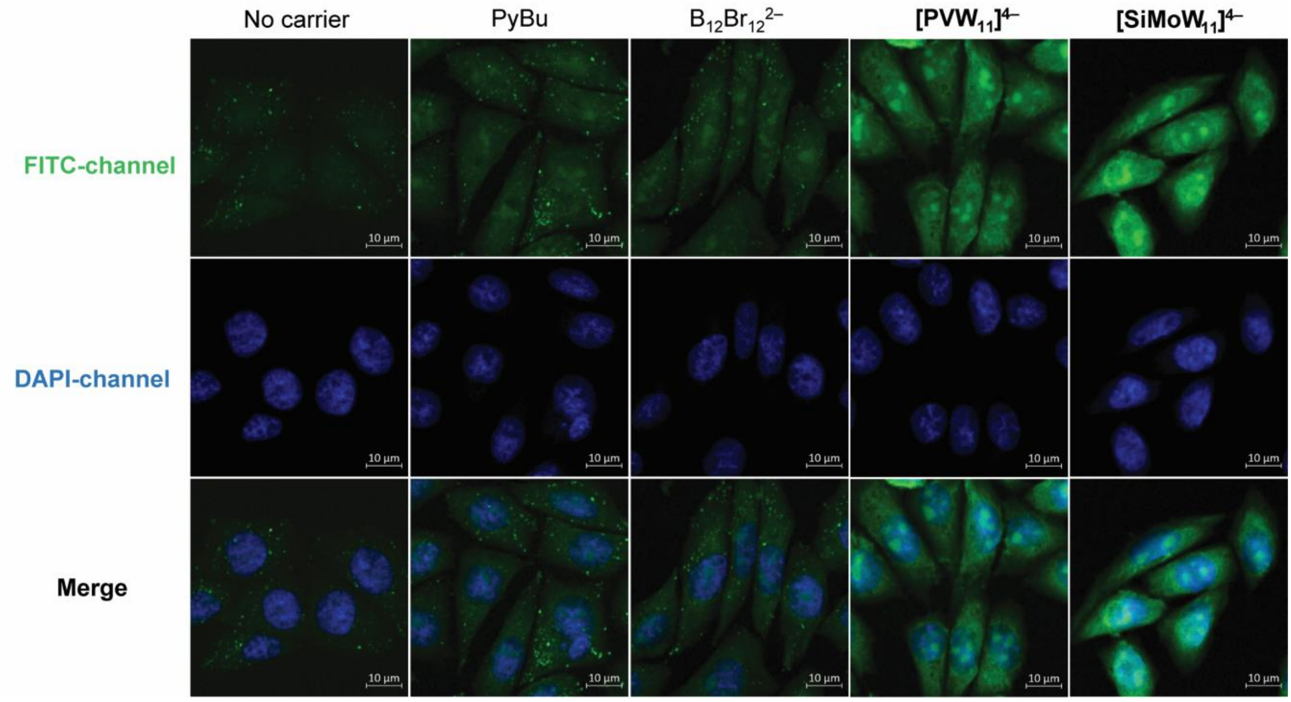

Figure S14. Representative confocal images of fixed CHO-K1 cells incubated with FITC-Arg_8 (4 μM) alone (no carrier) and promoted by the different carriers (5 μM). Inset scale bars: 10 μm. Representative images of two biological replicates.

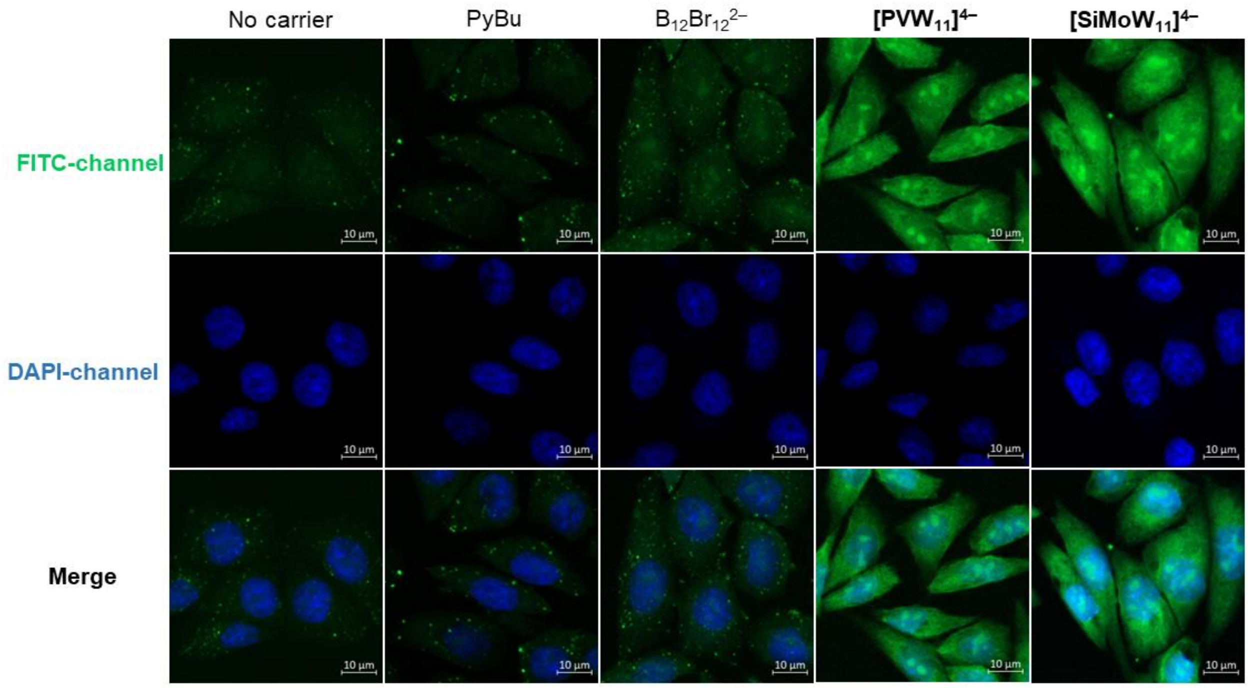

Figure S15. Representative confocal images of fixed CHO-K1 cells incubated with FITC-Arg_8 (4 µM) alone (no carrier) and promoted by the different carriers (10 µM). Inset scale bars: 10 µm. Representative images of two biological replicates.

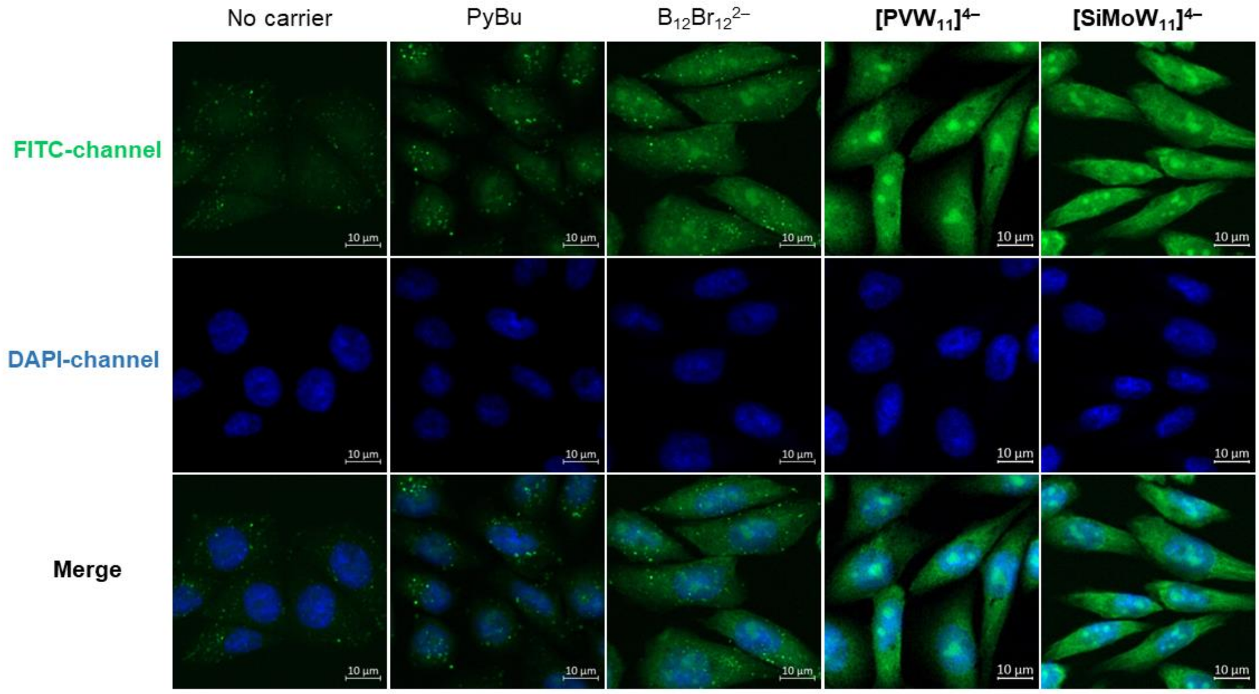

Figure S16. Representative confocal images of fixed CHO-K1 cells incubated with FITC-Arg_8 (4 µM) alone (no carrier) and promoted by the different carriers (50 µM). Inset scale bars: 10 µm. Representative images of two biological replicates.

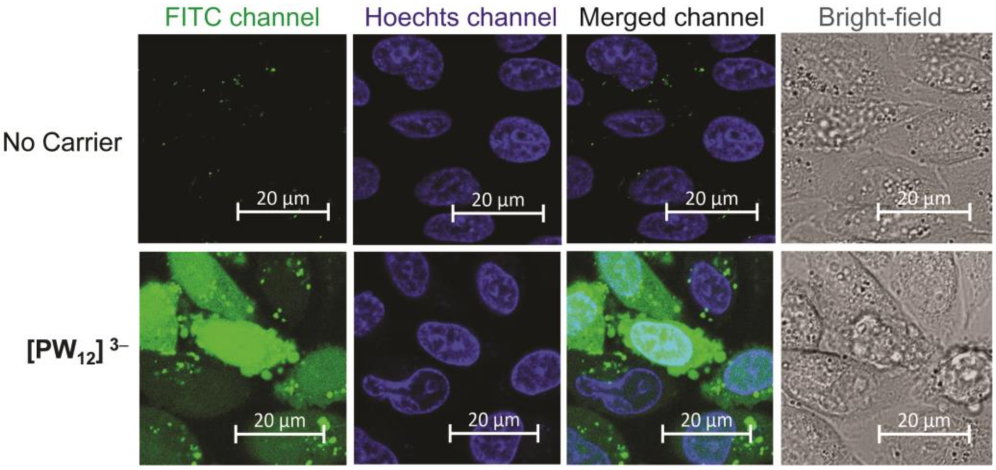

Figure S17. Representative confocal images of FITC-Arg$_8$ uptake (5 μM) alone (no carrier) and promoted by a solution of **[PW$_{12}$]$^{3-}$** (5 μM) in live CHO-K1 cells. Representative images of four biological replicates. Membrane blebbing shows that the cell morphology and intactness was highly compromised, which is attributed to membrane lysis by **[PW$_{12}$]$^{3-}$** or its hydrolysis products.

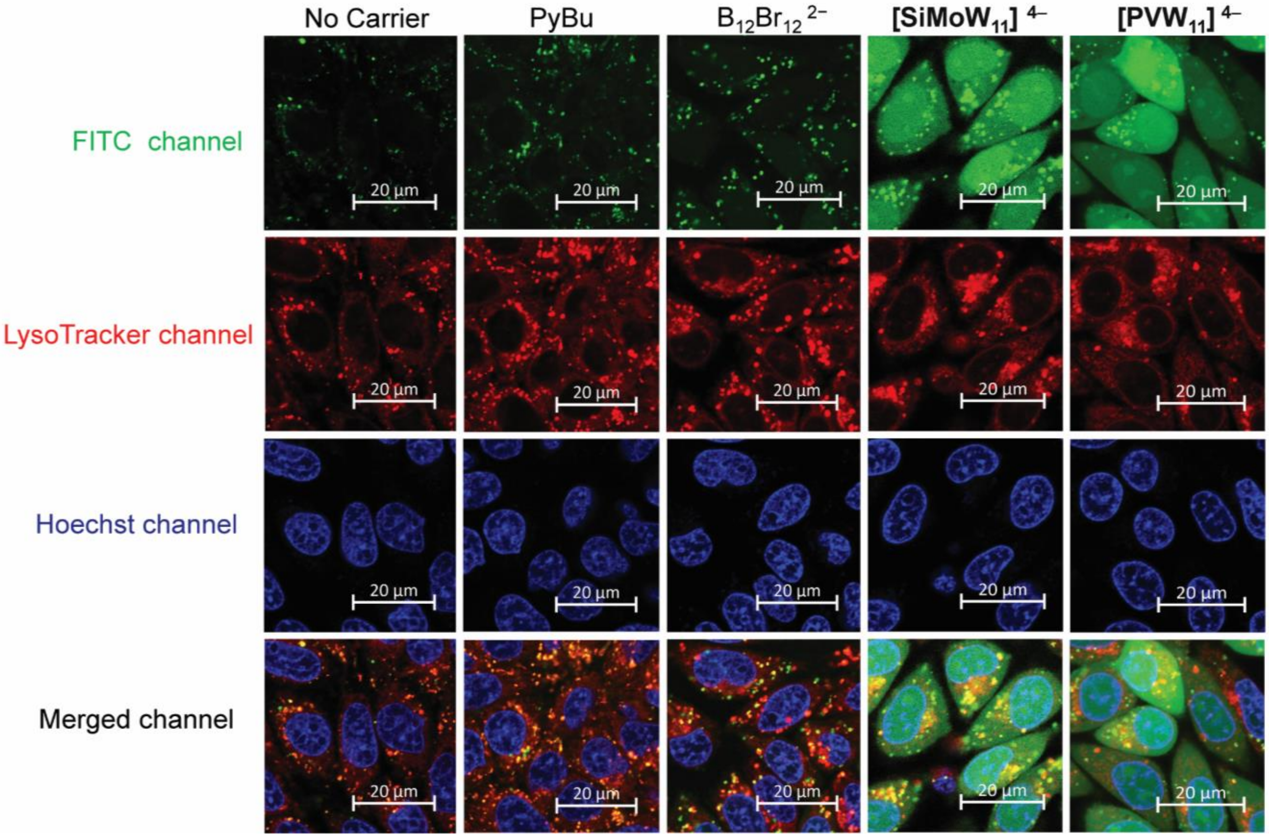

Figure S18. Representative confocal images of live CHO-K1 cells treated with 5 μM FITC-Arg$_8$ alone (no carrier) and in the presence of PyBu, $B_{12}Br_{12}^{2-}$, **[SiMoW$_{11}$]$^{4-}$**, and **[PVW$_{11}$]$^{4-}$** (5 μM, from left to right), and further incubated with Hoechst 33342 (10 μg/mL) and LysoTracker (1 μM), for nuclear and lysosomal staining, respectively. Representative images of two biological replicates.

7. IR spectroscopy

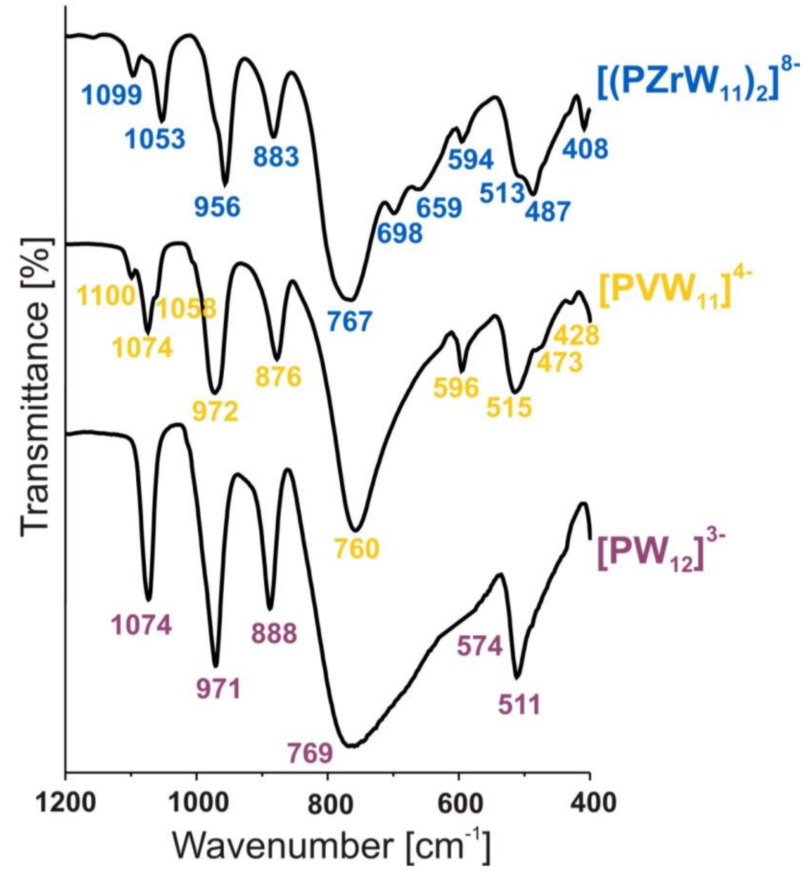

Figure S19. IR-spectra of three P-centered Keggin POTs – **$Na_3[P^VW^{VI}_{12}O_{40}]\cdot 8H_2O$**, **$K_4[\alpha\text{-}P^VV^VW^{VI}_{11}O_{40}]\cdot 4H_2O$**, **$(Et_2NH_2)_8[\{\alpha\text{-}P^VW^{VI}_{11}O_{39}Zr^{IV}(\mu\text{-}OH)(H_2O)\}_2]\cdot 7H_2O$** – in the fingerprint for POT anions range of 1200 - 400 cm^{-1}.

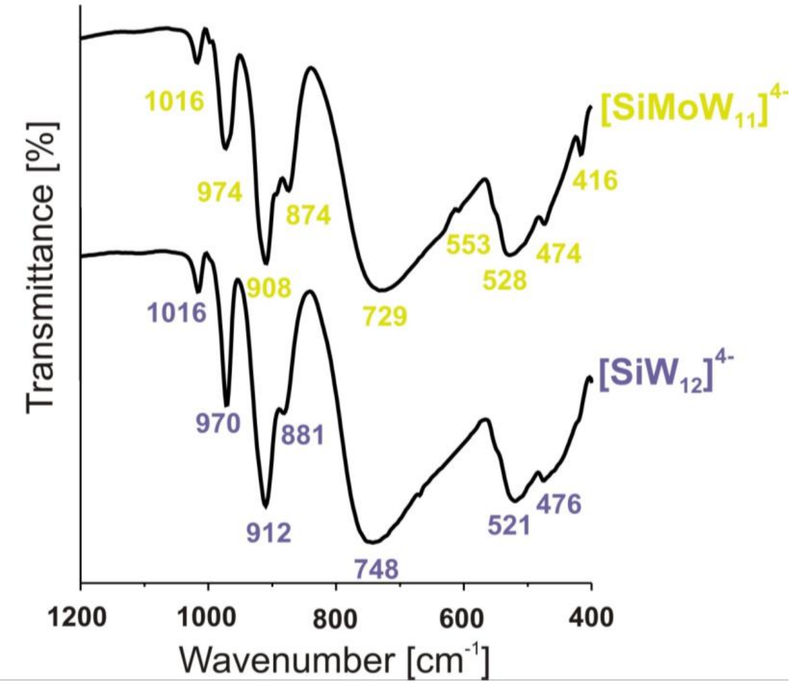

Figure S20. IR-spectra of Si-centered Keggin POTs – **$K_4[Si^{IV}W^{VI}_{12}O_{40}]\cdot 14H_2O$** and **$K_4[\alpha\text{-}Si^{IV}Mo^{VI}W^{VI}_{11}O_{40}]\cdot 24H_2O$** – in the fingerprint for POT anions range of 1200 - 400 cm^{-1}.

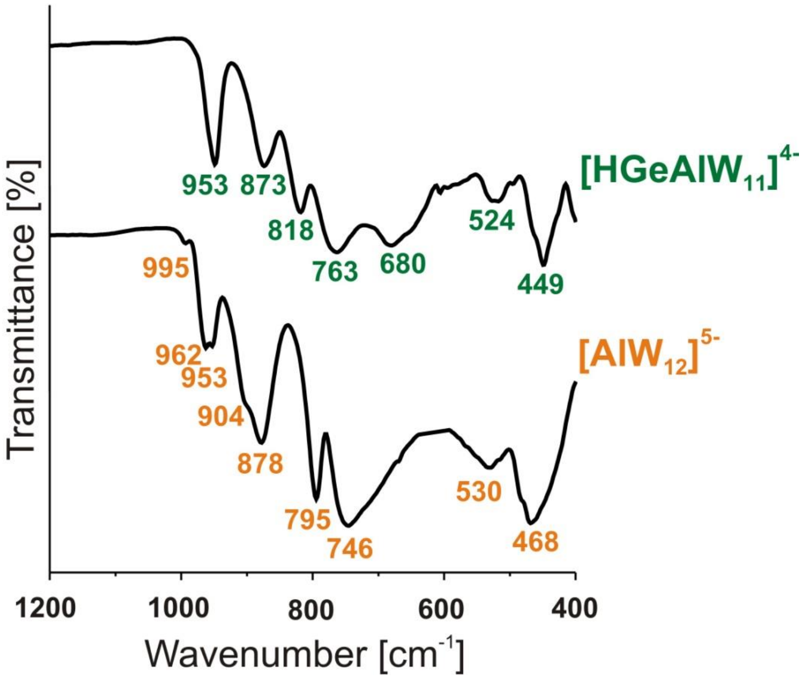

Figure S21. IR-spectra of Al- and Ge-centered Keggin POTs – **$(C_4H_{12}N)_4[HAl^{III}Ge^{IV}W^{VI}_{11}O_{39}(H_2O)]\cdot 11H_2O$** and **$Na_5[Al^{III}W^{VI}_{12}O_{40}]\cdot 12H_2O$** – in the fingerprint for POT anions range of 1200 - 400 cm^{-1}.

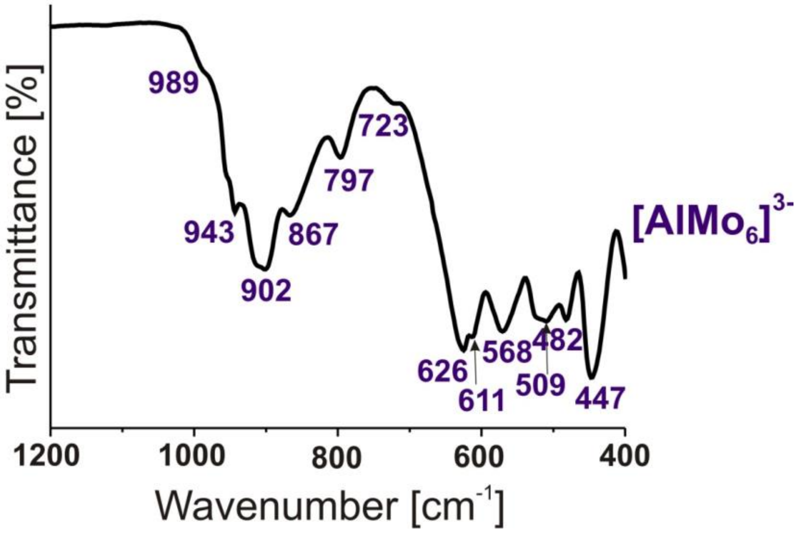

Figure S22. IR-spectrum of Anderson type **$Na_3[Al^{III}(OH)_6Mo^{VI}_6O_{18}]\cdot 2H_2O$** in the fingerprint range of 1200 - 400 cm^{-1}.

Table S7. Attribution and positions of the bands observed in IR-spectra of all tested POMs in the fingerprint region 1200-400 cm^{-1}.

POM	Bands [cm^{-1}]	Bands [cm^{-1}] according to literature data	Reference	Attribution
**$Na_3[P^{V}W^{VI}{}_{12}O_{40}]\cdot 8H_2O$**	1074	1080	[34]	$^{\nu}$P-O
	971	982		$^{\nu}$W=O
	888, 769, 574, 511	893, 798, 596, 526		$^{\nu}$W=O, $^{\delta}$W-O-W
**$K_4[\alpha\text{-}P^{V}V^{V}W^{VI}{}_{11}O_{40}]\cdot 4H_2O$**	1100, 1074, 1058	1099, 1076	[35]	$^{\nu}$P-O
	972	982		$^{\nu}$W=O
	876, 760, 596, 515, 473, 428	881, 784		$^{\nu}$W=O, $^{\delta}$W-O-W
**$(Et_2NH_2)_8[\{\alpha\text{-}P^{V}W^{VI}{}_{11}O_{39}Zr^{IV}(\mu\text{-}OH)(H_2O)\}_2]\cdot 7H_2O$**	1099, 1053	1098, 1057	[36]	$^{\nu}$P-O
	956	960		$^{\nu}$W=O
	883, 767, 698, 659, 594, 513, 487, 408	889, 810, 712, 670, 596, 514		$^{\nu}$W=O, $^{\delta}$W-O-W
**$K_4[Si^{IV}W^{VI}{}_{12}O_{40}]\cdot 14H_2O$**	1016	1020, 999	[37]	$^{\nu}$Si-O
	970	980, 940		$^{\nu}$W=O
	912, 881, 748, 521, 476	925, 894, 878, 780, 550, 530, 474, 373		$^{\nu}$W=O, $^{\delta}$W-O-W
**$K_4[\alpha\text{-}Si^{IV}Mo^{VI}W^{VI}{}_{11}O_{40}]\cdot 24H_2O$**	1016	1016	[35]	$^{\nu}$Si-O
	974	973		$^{\nu}$W=O
	908, 874, 729, 553, 528, 474, 416	920, 973, 877, 773		$^{\nu}$W=O, $^{\delta}$W-O-W
**$Na_5[Al^{III}W^{VI}{}_{12}O_{40}]\cdot 12H_2O$**	962, 953	955	[38]	$^{\nu}$W=O
	904, 878, 795, 746, 530, 468	883, 799, 758, 534, 498, 468		$^{\nu}$W=O, $^{\delta}$W-O-W
**$(C_4H_{12}N)_4[HAl^{III}Ge^{IV}W^{VI}{}_{11}O_{39}(H_2O)]\cdot 11H_2O$**	953	950	[39]	$^{\nu}$W=O
	873, 818, 763, 680, 524, 449	874, 820, 764, 677, 517, 447		$^{\nu}$W=O, $^{\delta}$W-O-W
**$Na_3[Al^{III}(OH)_6Mo^{VI}{}_6O_{18}]\cdot 2H_2O$**	943	947	[40]	$^{\nu}$W=O
	902, 867, 797, 723, 626, 611, 568, 509, 482, 447	920, 845, 650, 574, 530, 447		$^{\nu}$W=O, $^{\delta}$W-O-W

8. Thermogravimetric analysis

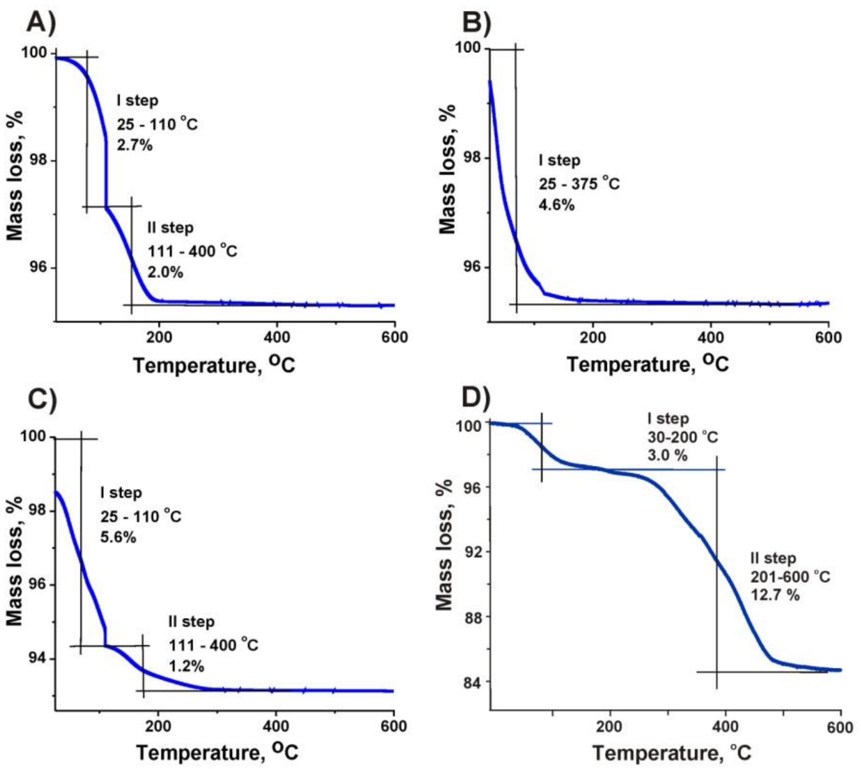

Figure S23a. Thermogravimetric curve of tested POMs: A) **$Na_3[P^{V}W^{VI}_{12}O_{40}]·8H_2O$**; B) **$K_4[Si^{IV}W^{VI}_{12}O_{40}]·14H_2O$**; C) **$Na_5[Al^{III}W^{VI}_{12}O_{40}]·12H_2O$**; D) **$(C_4H_{12}N)_4[HAl^{III}Ge^{IV}W^{VI}_{11}O_{39}(H_2O)]·11H_2O$**, in the temperature region 25 – 600 ºC with a heating rate of 5 ºC min^{-1}.

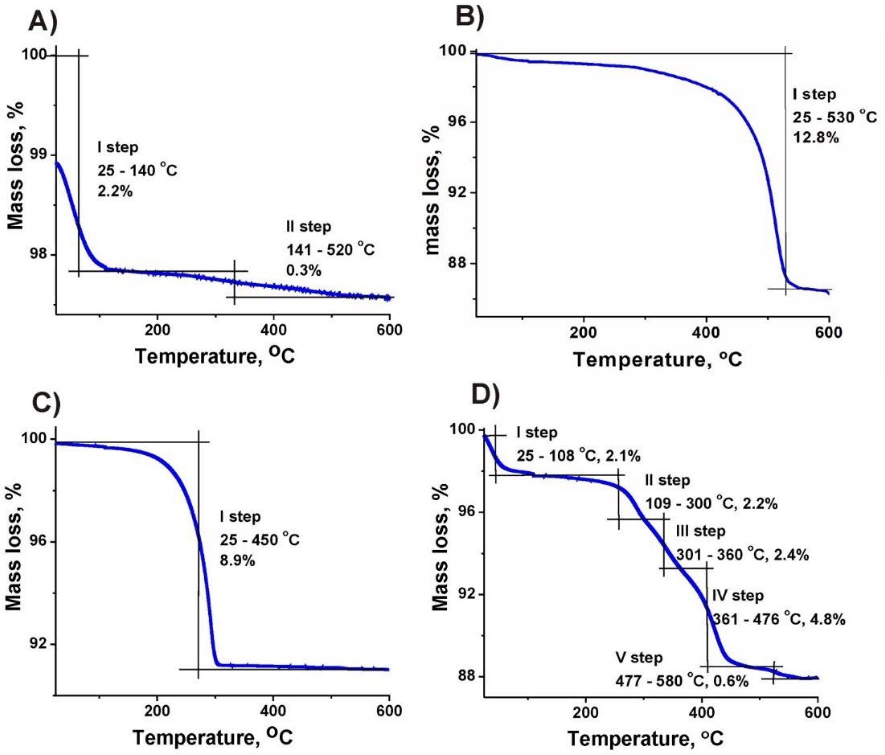

Figure S23b. Thermogravimetric curve of tested POMs: A) **$K_4[\alpha\text{-}P^{V}V^{V}W^{VI}_{11}O_{40}]·4H_2O$**; B) **$K_4[\alpha\text{-}Si^{IV}Mo^{VI}W^{VI}_{11}O_{40}]·24H_2O$**; C) **$(Et_2NH_2)_8[\{\alpha\text{-}P^{V}W^{VI}_{11}O_{39}Zr^{IV}(\mu\text{-}OH)(H_2O)\}_2]·7H_2O$**; D) **$Na_3[Al^{III}(OH)_6Mo^{VI}_6O_{18}]·2H_2O$**, in the temperature region 25 – 600 ºC with a heating rate of 5 ºC min^{-1}.

Table S8. The mass-loss steps with corresponding assignment to the solvent molecules and /or counter-cations for all tested POMs.

Compound	Figure	Step	T, °C	mass-loss, %	Number and kind of molecules corresponding to mass-loss
$Na_3[P^VW^{VI}_{12}O_{40}]\cdot 8H_2O$	S23aA	I	25 – 110	2.7	4.5 H_2O
		II	111 – 400	2.0	3.5 H_2O
$K_4[Si^{IV}W^{VI}_{12}O_{40}]\cdot 14H_2O$	S23aB	I	25 – 375	4.6	8 H_2O
$Na_5[Al^{III}W^{VI}_{12}O_{40}]\cdot 12H_2O$	S23aC	I	25 – 110	5.6	10 H_2O
		II	111 – 400	1.2	2 H_2O
$(C_4H_{12}N)_4[HAl^{III}Ge^{IV}W^{VI}_{11}O_{39}(H_2O)]\cdot 11H_2O$	S23aD	I	30 – 200	3.0	5.5 H_2O
		II	201 – 600	12.7	6.5 H_2O + 4 TMA
$K_4[\alpha\text{-}P^VV^VW^{VI}_{11}O_{40}]\cdot 4H_2O$	S23bA	I	25 – 140	2.2	3.5 H_2O
		II	141 – 520	0.3	0.5 H_2O
$K_4[\alpha\text{-}Si^{IV}Mo^{VI}W^{VI}_{11}O_{40}]\cdot 24H_2O$	S23bB	I	25 – 530	12.8	24 H_2O
$Na_3[Al^{III}(OH)_6Mo^{VI}_6O_{18}]\cdot 2H_2O$	S23bC	I	25 – 450	8.9	5 H_2O (3 H_2O from 6 OH^- in anion)
$(Et_2NH_2)_8[\{\alpha\text{-}P^VW^{VI}_{11}O_{39}Zr^{IV}(\mu\text{-}OH)(H_2O)\}_2]\cdot 7H_2O$	S23bD	I	25 – 108	2.1	7 H_2O
		II	109 – 300	2.2	2 Et_2NH_2
		III	301 – 360	2.4	2 Et_2NH_2
		IV	361 – 476	4.8	4 Et_2NH_2
		V	477 – 580	0.6	2 H_2O

9. NMR-spectroscopic characterization of identity and stability

NMR spectroscopy for all POMs tested was performed under experimental conditions. Since the choice of buffer for transport experiments depends on the stability of the anion, **$[SiW_{12}]^{4-}$**, **$[AlW_{12}]^{5-}$**, **$[GeAlW_{11}]^{5-}$**, **$[AlMo_6]^{3-}$**, **$[(PZrW_{11})_2]^{8-}$** were tested under neutral conditions (10 mM Hepes or Tris-HCl, 107 mM NaCl, pH7.5), **$[PVW_{11}]^{4-}$** and **$[SiMoW_{11}]^{4-}$** in both weakly acidic (10 mM Mes, 107 mM NaCl, pH 5.5) and neutral buffer (10 mM Hepes or Tris, 107 mM NaCl, pH7.5), and **$[PW_{12}]^{3-}$** only in acidic buffer (10 mM Mes, 107 mM NaCl, pH 5.5).

For the stability studies of **$[SiW_{12}]^{4-}$** only ^{183}W NMR spectroscopy was applied. Considering the very low abundance of the ^{29}Si isotope (4.67 %), we were unable to obtain reasonable data for **$[SiW_{12}]^{4-}$** solutions. We therefore chose ^{183}W NMR as an established and more accessible method. Since the abundance of ^{183}W isotope is also relatively low (14.3 %), the acquisition takes 60 h and the POM concentration should not be lower than 60 mg/mL. Therefore, stability studies on 3 mM POT solutions in 10 mM Tris-HCl buffer were not possible.

For compounds that are unstable under experimental conditions, spectra are shown under conditions under which they are stable to confirm their purity.

Table S9. Results of stability investigation by NMR spectroscopy.

POM	Buffer/Media	Figure	^{31}P, ^{51}V, ^{183}W or ^{27}Al shifts, ppm	POM anions with % for parent (active) anion based on signals integration	Peak assignment to ref.
**$[PVW_{11}]^{4-}$**	D_2O	S24A, B	**^{31}P**: –14.8; **^{51}V**: –556	**$[P^VV^VW^{VI}_{11}O_{40}]^{4-}$**	[35]
	Mes pH 5.5 freshly prepared	S25A	**^{31}P**: –10.8 and –14.9	$[P^VW^{VI}_{11}O_{39}]^{7-}$ (1%) and $[P^VV^VW^{VI}_{11}O_{40}]^{4-}$ (99%)	[35,41]
	Mes pH 5.5 after 1 day	S25B	**^{31}P**: –10.8 and –14.9	$[P^VW^{VI}_{11}O_{39}]^{7-}$ (1%) and **$[P^VV^VW^{VI}_{11}O_{40}]^{4-}$** (99%)	[35,41]
	Mes pH 5.5 after 5 days	S25C	**^{31}P**: –10.8 and –14.9	$[P^VW^{VI}_{11}O_{39}]^{7-}$ (1%) and **$[P^VV^VW^{VI}_{11}O_{40}]^{4-}$** (99%)	[35,41]
	Tris-HCl pH 7.5 freshly prepared	S26A	**^{31}P**: –11.4, –14.4 and –14.9	$[P^VW^{VI}_{11}O_{39}]^{7-}$ and **$[P^VV^VW^{VI}_{11}O_{40}]^{4-}$** (81%)	[35,41]
	Tris-HCl pH 7.5 after 1 day	S26B	**^{31}P**: –11.4, –14.4 and –14.9	$[P^VW^{VI}_{11}O_{39}]^{7-}$ and **$[P^VV^VW^{VI}_{11}O_{40}]^{4-}$** (71%)	[35,41]
	Tris-HCl pH 7.5 after 5 days	S26C	**^{31}P**: –11.4, –14.4 and –14.9	$[P^VW^{VI}_{11}O_{39}]^{7-}$ and **$[P^VV^VW^{VI}_{11}O_{40}]^{4-}$** (68%)	[35,41]
	Nutrient mixture F-12 Ham[a)] freshly prepared	S28A, S29A	**^{31}P**: 0.4 (free phosphate from medium), –10.6, –10.7, –10.8, –11.1 and –14.9; **^{51}V**: –509.2, –558.8	$[P^VW^{VI}_{11}O_{39}]^{7-}$ (coordinated to different alkali and earth alkali metals leading to different shifts) and **$[P^VV^VW^{VI}_{11}O_{40}]^{4-}$** (43%)	[35,41]
	Nutrient mixture F-12 Ham[a)] after incubation for 1 h at 37 °C	S28B, S29B	**^{31}P**: 0.4 (free phosphate from medium), –10.1, –10.8, –13.7, –14.4 and –14.9; **^{51}V**: –509.2, –523.1, –526.7, –554.2, –558.8, –559.7	$[P^VW^{VI}_{11}O_{39}]^{7-}$ (coordinated to different alkali and earth alkali metals leading to different shifts) and **$[P^VV^VW^{VI}_{11}O_{40}]^{4-}$** (16%)	[35,41]
**$[SiMoW_{11}]^{4-}$**	Mes pH 5.5	S30A	**^{183}W**: –99.9, –100.2, –102.4, –103.0, –103.5, –107.4	**$[Si^{IV}Mo^{VI}W^{VI}_{11}O_{40}]^{4-}$**	[35]
	Tris-HCl pH 7.5	S30B	**^{183}W**: –99.3, –99.6, –101.8, –102.8, –106.7	**45% $[Si^{IV}Mo^{VI}W^{VI}_{11}O_{40}]^{4-}$** (based on the signal intensities; the signal at –102.4 ppm becomes more pronounced suggesting an overlap with a transformation product, presumably $[Si^{IV}W^{VI}_{12}O_{40}]^{4-}$)	[35]
	Nutrient mixture F-12 Ham after incubation for 1 h at 37 °C	S30C	**^{183}W**: –99.4, –99.7, –101.9, –102.5, –102.9, –106.9	**50% $[Si^{IV}Mo^{VI}W^{VI}_{11}O_{40}]^{4-}$** (based on the signal intensities; the signal at –102.4 ppm becomes more pronounced suggesting an overlap with a transformation product, presumably $[Si^{IV}W^{VI}_{12}O_{40}]^{4-}$)	[35]
**$[AlMo_6]^{3-}$**	Hepes pH 7.5 freshly prepared	S31A	**^{27}Al**: 16.3	**$[Al^{III}(OH)_6Mo^{VI}_6O_{18}]^{3-}$**	[40]
	Hepes pH 7.5 after 1 day	S31B	**^{27}Al**: 16.3	**$[Al^{III}(OH)_6Mo^{VI}_6O_{18}]^{3-}$**	[40]
**$[AlW_{12}]^{5-}$**	Tris-HCl pH 7.5 freshly prepared	S32A	**^{27}Al**: 72.6	**$[Al^{III}W^{VI}_{12}O_{40}]^{5-}$**	[38]
	Tris-HCl pH 7.5 after 1 day	S32B	**^{27}Al**: 72.6	**$[Al^{III}W^{VI}_{12}O_{40}]^{5-}$**	[38]
**$[GeAlW_{11}]^{5-}$**	Tris-HCl pH 7.5 freshly prepared	S33A	**^{27}Al**: 9.4	**$[HAl^{III}Ge^{IV}W^{VI}_{11}O_{39}(H_2O)]^{4-}$**	[39]
	Tris-HCl pH 7.5 after 1 day	S33B	**^{27}Al**: 9.4	**$[HAl^{III}Ge^{IV}W^{VI}_{11}O_{39}(H_2O)]^{4-}$**	[39]
**$[(PZrW_{11})_2]^{8-}$**	Hepes pH 7.5 freshly prepared	S34A	**^{31}P**: –13.7, –14.8, –14.9	**$[\{\alpha\text{-}PW_{11}O_{39}Zr(\mu\text{-}OH)(H_2O)\}_2]^{8-}$** (99%) and $[Zr(\alpha\text{-}PW_{11}O_{39})_2]^{10-}$	[30]
	Hepes pH 7.5 after 1 day	S34B	**^{31}P**: –13.7, –14.8, –14.9	**$[\{\alpha\text{-}PW_{11}O_{39}Zr(\mu\text{-}OH)(H_2O)\}_2]^{8-}$** (99%) and $[Zr(\alpha\text{-}PW_{11}O_{39})_2]^{10-}$	[30]
**$[PW_{12}]^{3-}$**	NaOAc/HOAc pH 4 freshly prepared	S35A	**^{31}P**: –12.4, –13.1, –13.7; –15.2	**$[P^VW^{VI}_{12}O_{40}]^{3-}$** (30%) and intermediate anions	[41]
	NaOAc/HOAc pH 4 after 1 day	S35B	**^{31}P**: –12.4, –13.1, –13.7; –15.2	**$[P^VW^{VI}_{12}O_{40}]^{3-}$** (28%) and intermediate anions	[41]
	Mes pH 5.5 freshly prepared	S36A	**^{31}P**: –11.0, –12.4; 13.1, –13.7; –14.7	**$[P^VW^{VI}_{12}O_{40}]^{3-}$** (4%), $[P^VW^{VI}_{11}O_{39}]^{7-}$ (61%) and intermediate anions	[41]
	Mes pH 5.5 after 1 day	S36B	**^{31}P**: –11.0, –12.4; 13.1; –13.3; –13.9; –14.7	**$[P^VW^{VI}_{12}O_{40}]^{3-}$** (5%), $[P^VW^{VI}_{11}O_{39}]^{7-}$ (61%) and intermediate anions	[41]
**$[SiW_{12}]^{4-}$**	Hepes pH 7.5	S37	**^{183}W**: –99.8; –100.8; –114.5; –119.8; –127.8; –140.4; –174.6	**$[Si^{IV}W^{VI}_{12}O_{40}]^{4-}$** and $[Si^{IV}W^{VI}_{11}O_{39}]^{8-}$ (high noise to signal ratio, correct integration of peak cannot be performed; but clearly $[Si^{IV}W^{VI}_{11}O_{39}]^{8-}$ is predominant)	[35]

[a)] According to the provider Sigma-Aldrich, Nutrient Mixture F-12 Ham for cell culturing contains sodium pyruvate (0.11 g/L), phenol red, L-glutamine, and does not contain $NaHCO_3$ and Hepes, for more details please see https://www.sigmaaldrich.com/AT/en/technical-documents/technical-article/cell-culture-and-cell-culture-analysis/mammalian-cell-culture/f-12-Ham.

9.1. ^{31}P and ^{51}V NMR spectra of $[PVW_{11}]^{4-}$

Stability of $[PVW_{11}]^{4-}$ in 10 mM Mes buffer pH 5.5: ^{31}P NMR studies were firstly performed on 3 mM solutions of $[\alpha\text{-}P^{V}V^{V}W^{VI}_{11}O_{40}]^{4-}$ in 10 mM Mes buffer pH 5.5 (Figure S25). The ^{31}P NMR recorded immediately (Figure S24A), one day (Figure S25B), and five days (Figure S25C) after solution preparation display the same signals that can be attributed to the intact $[\alpha\text{-}P^{V}V^{V}W^{VI}_{11}O_{40}]^{4-}$ (–14.9 ppm) and the monolacunary $[\alpha\text{-}P^{V}W^{VI}_{11}O_{40}]^{7-}$ (–10.8 ppm) species formed upon partial hydrolysis. The final pH upon the POM's dissolution amounts to 4.9 and remains unchanged indicating the stability of the immediately formed species for up to five days. The integration of the ^{31}P signals suggests the presence of monolacunary anion $[\alpha\text{-}P^{V}W^{VI}_{11}O_{40}]^{7-}$ as about 1 % on the day of preparation.

Stability of $[PVW_{11}]^{4-}$ 10 mM Tris-HCl buffer pH 7.4: In 10 mM Tris-HCl buffer the solution's pH decreases from pH 7.5 to 5.4, but $[\alpha\text{-}PVW_{11}O_{40}]^{4-}$ is present in sufficient quantity (around 81 % based on ^{31}P signals integration, Figure S26).

Stability of $[PVW_{11}]^{4-}$ in Ham's medium pH 7.4: The cellular uptake with the cell line CHO-K1 was performed in nutrient mixture F-12 Ham (pH 7.4), where the stability of $\mathbf{[PVW_{11}]^{4-}}$ has been also tested (Figure S28-S29). In the freshly prepared solution, the concentration of $[\alpha\text{-}P^{V}V^{V}W^{VI}_{11}O_{40}]^{4-}$ is 43 %, but after incubation for 1 h at 37 °C, it decreases to about 16%.

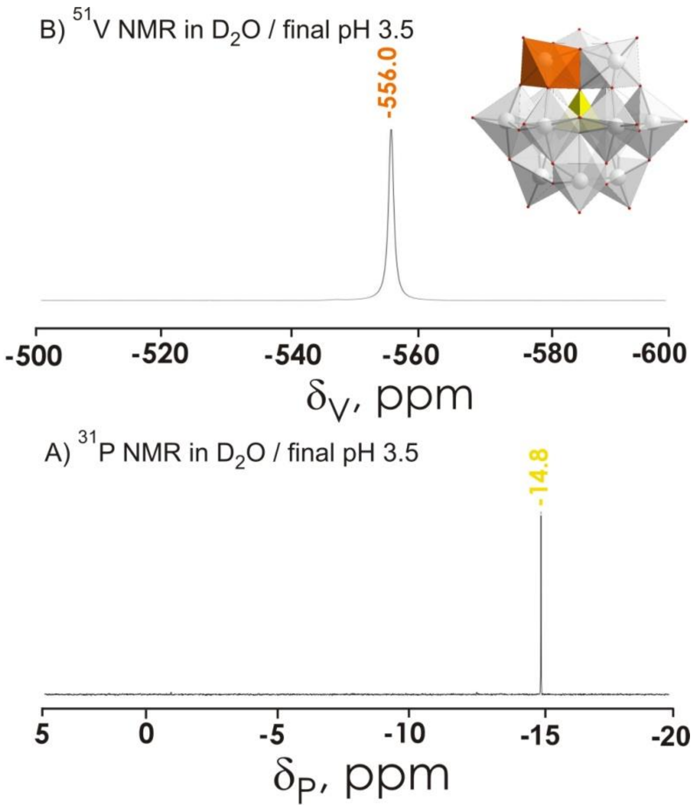

Figure S24. ^{31}P (A) and ^{51}V (B) NMR spectra of 3 mM solutions of $\mathbf{[PVW_{11}]^{4-}}$ recorded in D_2O. In polyhedral representation of $[P^{V}V^{V}W^{VI}_{11}O_{40}]^{4-}$ $\{VO_6\}$ is shown in orange and $\{PO_4\}$ in yellow matching shifts assignment with the same color in A) and B). For signal assignment see Table S9.

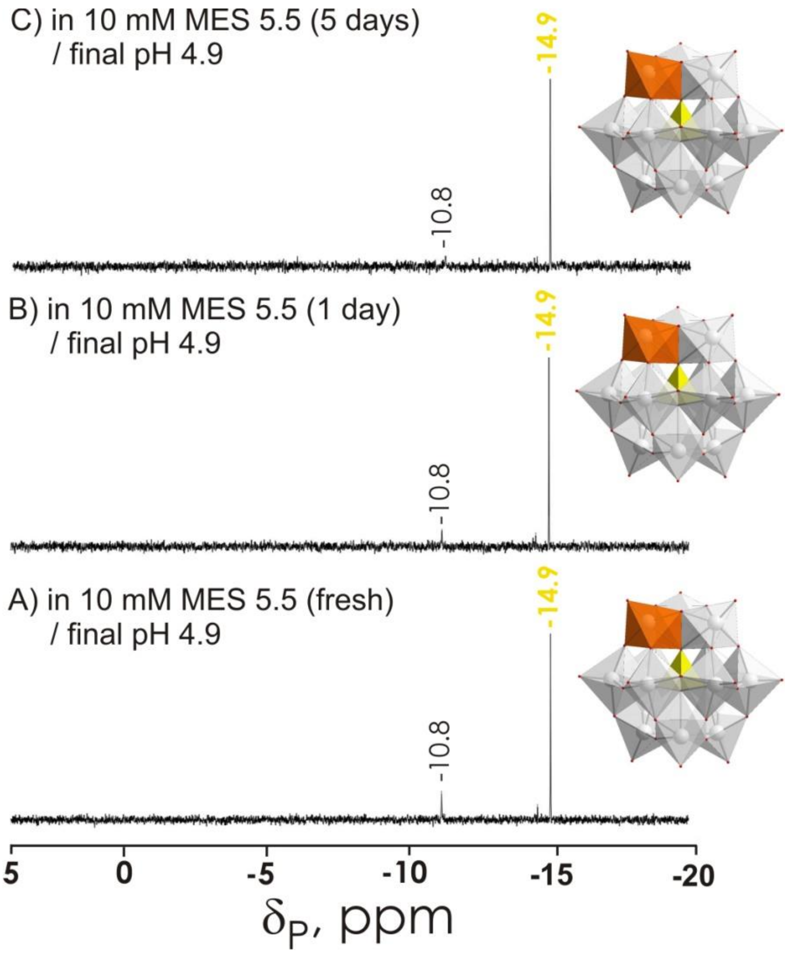

Figure S25. ^{31}P NMR spectra of 3 mM solutions of **$[PVW_{11}]^{4-}$** recorded in A) 10 mM Mes pH 5.5 buffer on the day of preparation (appr. 3 h after POM dissolution); B) 10 mM Mes pH 5.5 buffer one day after preparation (appr. 26 h after POM dissolution); C) 10 mM Mes pH 5.5 buffer five days after preparation. In polyhedral representation of $[P^VV^VW^{VI}_{11}O_{40}]^{4-}$ $\{PO_4\}$ is shown in yellow matching shifts assignment with the same color in A), B) and C). Color code: $\{WO_6\}$, transparent grey; $\{PO_4\}$, yellow; $\{VO_6\}$, orange; O, red. For signal assignment see Table S9.

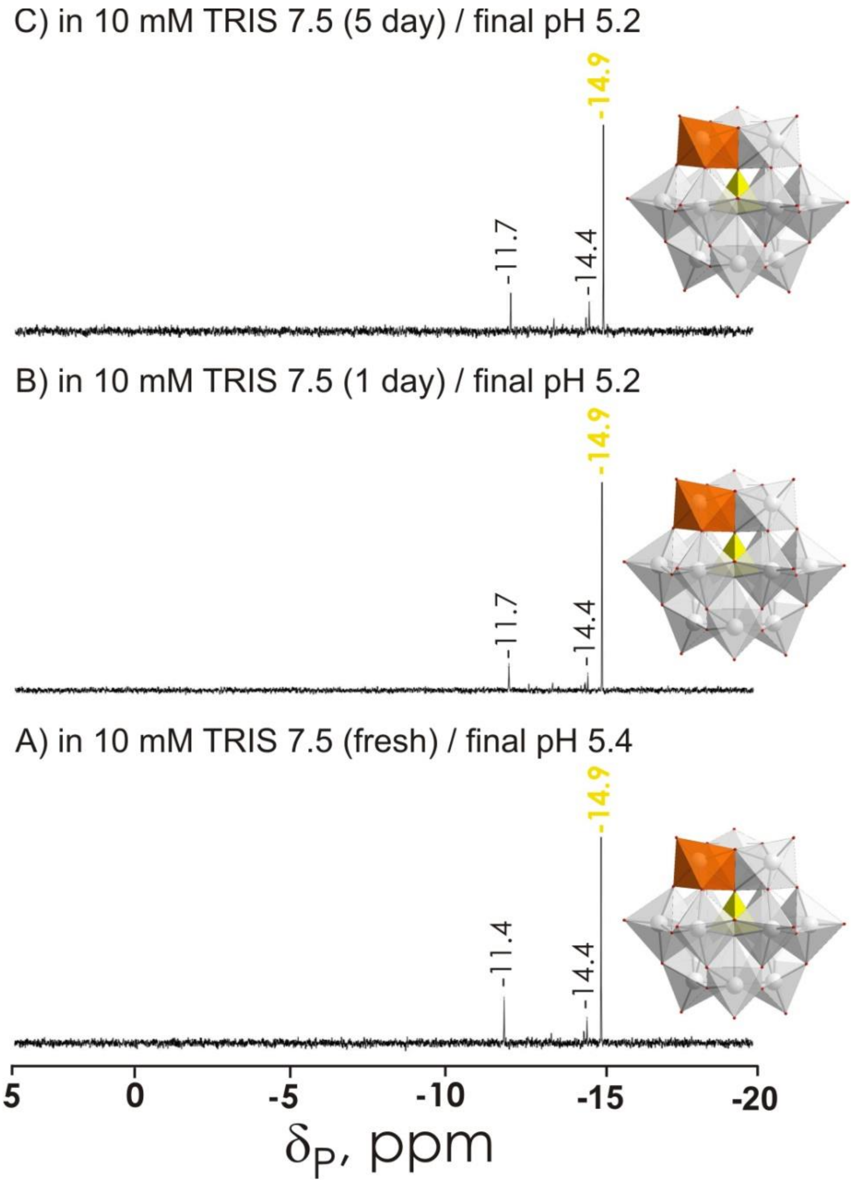

Figure S26. ^{31}P NMR spectra of 3 mM solutions of **$[PVW_{11}]^{4-}$** recorded in A) 10 mM Tris-HCl pH 7.5 buffer on the day of preparation (appr. 3 h after POM dissolution); B) 10 mM Tris-HCl pH 7.5 buffer one day after preparation (appr. 26 h after POM dissolution); C) 10 mM Tris-HCl pH 7.5 buffer five days after preparation. In polyhedral representation of $[P^{V}V^{V}W^{VI}_{11}O_{40}]^{4-}$ {PO_4} is shown in yellow matching shifts assignment with the same color in A), B) and C). Color code: {WO_6}, transparent grey; {PO_4}, yellow; {VO_6}, orange; O, red. For signal assignment see Table S9.

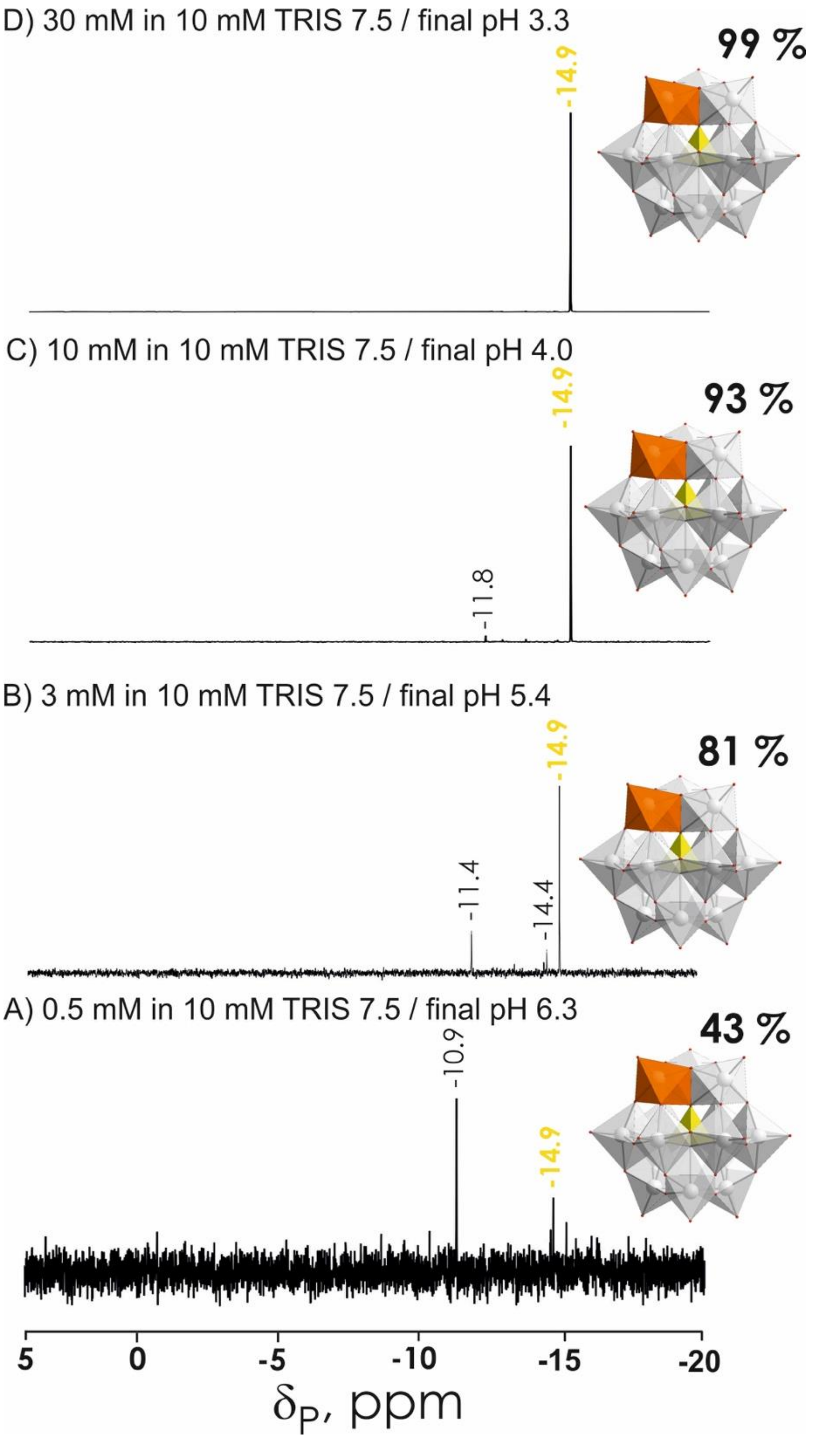

Figure S27. ^{31}P NMR spectra of 0.5 (A), 3 (B), 10 (C) and 30 (D) mM solutions of **$[PVW_{11}]^{4-}$** recorded in 10 mM Tris-HCl pH 7.5 buffer on the day of preparation (appr. 3 h after POM dissolution). In the polyhedral representation of $[P^{V}V^{V}W^{VI}_{11}O_{40}]^{4-}$ $\{PO_4\}$ matching shifts assignments are shown in yellow with the same color in A), B), and C). Color code: $\{WO_6\}$, transparent grey; $\{PO_4\}$, yellow; $\{VO_6\}$, orange; O, red. For signal assignment see Table S9.

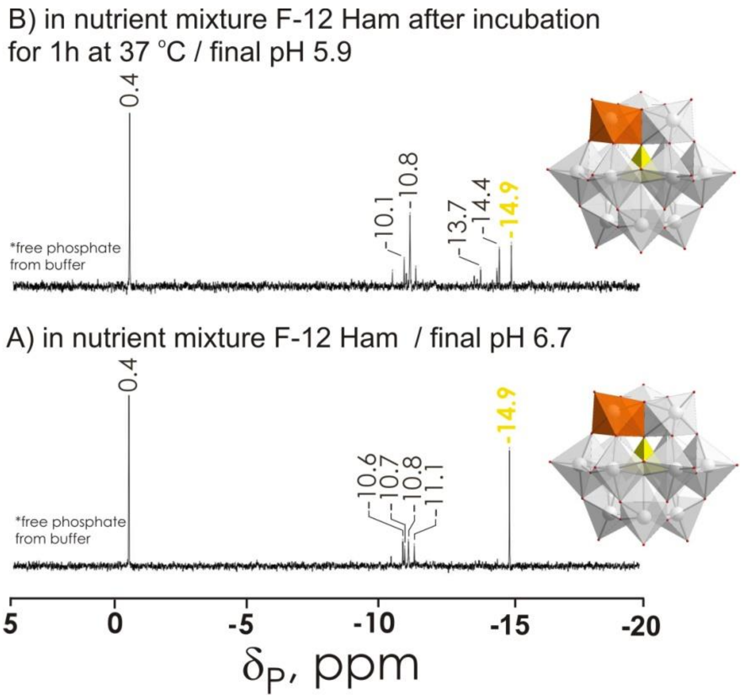

Figure S28. ${}^{31}P$ NMR spectra of 3 mM solutions of $\mathbf{[PVW_{11}]^{4-}}$ recorded in A) nutrient mixture F-12 Ham immediately after preparation; B) nutrient mixture F-12 Ham (for more details please see https://www.sigmaaldrich.com/AT/en/technical-documents/technical-article/cell-culture-and-cell-culture-analysis/mammalian-cell-culture/f-12-ham) after incubation for 1h at 37 °C. In polyhedral representation of $[P^{V}V^{V}W^{VI}{}_{11}O_{40}]^{4-}$ $\{PO_4\}$ is shown in yellow matching shifts assignment with the same color in A) and B). Color code: $\{WO_6\}$, transparent grey; $\{PO_4\}$, yellow; $\{VO_6\}$, orange; O, red. For signal assignment see Table S9.

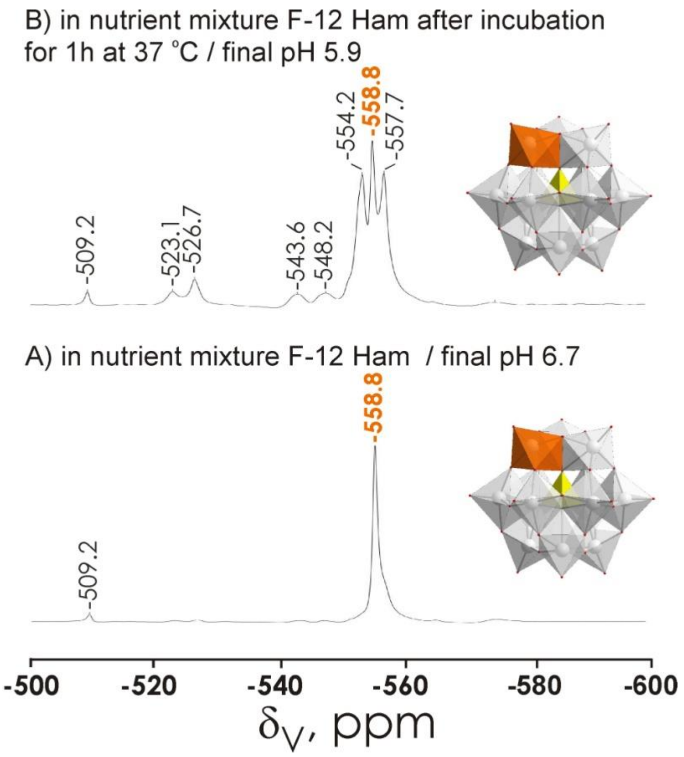

Figure S29. ${}^{51}V$ NMR spectra of 3 mM solutions of $\mathbf{[PVW_{11}]^{4-}}$ recorded in A) nutrient mixture F-12 Ham immediately after preparation; B) nutrient mixture F-12 Ham (for more details please see https://www.sigmaaldrich.com/AT/en/technical-documents/technical-article/cell-culture-and-cell-culture-analysis/mammalian-cell-culture/f-12-ham) after incubation for 1h at 37 °C. In polyhedral representation of $[P^{V}V^{V}W^{VI}{}_{11}O_{40}]^{4-}$ $\{VO_6\}$ is shown in orange matching shifts assignment with the same color in A) and B). Color code: $\{WO_6\}$, transparent grey; $\{PO_4\}$, yellow; $\{VO_6\}$, orange; O, red. For signal assignment see Table S9.

9.2. ^{183}W NMR spectra of $[SiMoW_{11}]^{4-}$

Stability of $[SiMoW_{11}]^{4-}$ in 33 mM Mes buffer pH 5.5: For the stability studies for $[\alpha\text{-}Si^{IV}Mo^{VI}W^{VI}_{11}O_{40}]^{4-}$ ^{29}Si, ^{95}Mo and ^{183}W NMR spectroscopy can be applied. Considering the very low abundance of the ^{29}Si isotope (4.67 %), we were unable to obtain reasonable data for $[\alpha\text{-}Si^{IV}Mo^{VI}W^{VI}_{11}O_{40}]^{4-}$ solutions. We therefore chose ^{183}W NMR as an established and more accessible method, and the **$[SiMoW_{11}]^{4-}$** spectrum was recorded in Mes pH 5.5 buffer (Figure S30A). Since the abundance of ^{183}W isotope is also relatively low (14.3 %), the acquisition takes 60 h and the POT concentration should not be lower than 60 mg/mL. The spectrum of **$[SiMoW_{11}]^{4-}$** shows only signals attributed to the intact $[\alpha\text{-}Si^{IV}Mo^{VI}W^{VI}_{11}O_{40}]^{4-}$ confirming its solution stability for up to 60 h (time of the spectrum acquisition).

Stability of $[SiMoW_{11}]^{4-}$ in 33 mM Tris-HCl buffer pH 7.4: The ^{183}W NMR spectrum of **$[SiMoW_{11}]^{4-}$** in Tris-HCl (Figure S30B) shows the same 5 signals at –99.3, –99.6, –101.8, –102.8 and –106.7 as in case of Mes pH 5.5, missing one signal at –103 ppm, which might be just too close to noise. The signal at –102.4 became too intense, pointing to possible re-arrangements of the POM to a more symmetrical product such as $[Si^{IV}W^{VI}_{12}O_{40}]^{4-}$.

Stability of $[SiMoW_{11}]^{4-}$ in Ham's medium pH 7.4: The spectrum recorded in a nutrient mixture F-12 Ham after incubation for 1 h at 37 °C (Figure S30C) indicates partial (~ 50%) integrity of $[\alpha\text{-}Si^{IV}Mo^{VI}W^{VI}_{11}O_{40}]^{4-}$.

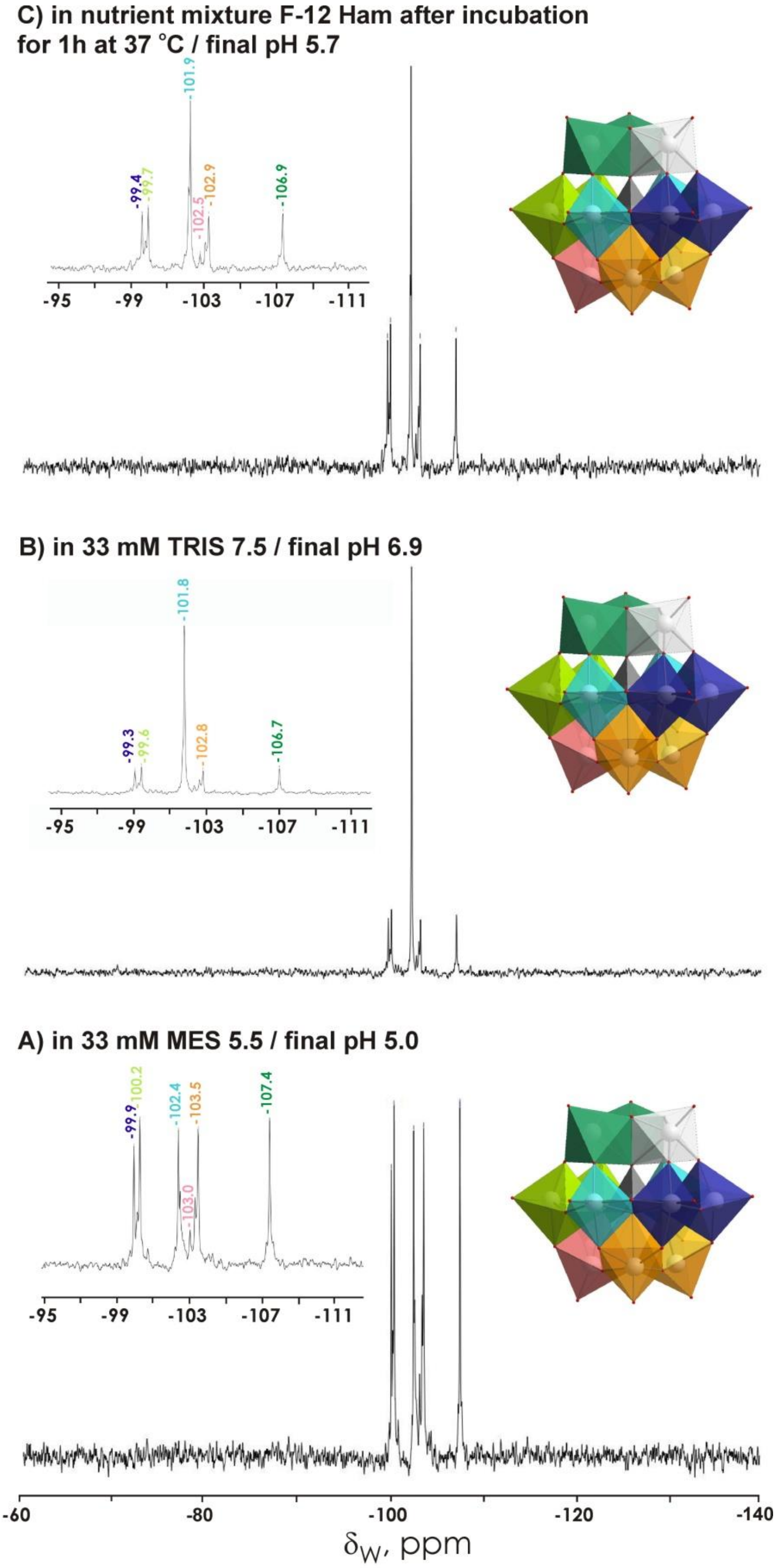

Figure S30. ^{183}W NMR spectrum of a 10 mM solution of $K_4[\alpha\text{-}SiMoW_{11}O_{40}]\cdot 16H_2O$ recorded in A) 33 mM Mes pH 5.5 buffer (final pH is 5); B) 33 mM TRIS pH 7.5 buffer (final pH is 6.9); C) nutrient mixture F-12 Ham (for more details please see https://www.sigmaaldrich.com/AT/en/technical-documents/technical-article/cell-culture-and-cell-culture-analysis/mammalian-cell-culture/f-12-ham) after the incubation for 1 h at 37 °C (final pH 5.7). In polyhedral representation of $[Si^{IV}Mo^{VI}W^{VI}_{11}O_{40}]^{4-}$ six types of $\{WO_6\}$ are shown in six different colors matching shifts assignment with the same color in A), B) and C). The signal at −102.4 ppm becomes more pronounced in the presence of Tris-HCl and the nutrient mixture F-12 Ham, suggesting an overlap with a decomposition product, presumably $[Si^{IV}W^{VI}_{12}O_{40}]^{4-}$. Color code: $\{WO_6\}$, green, lime, indigo, blue, pink, and orange; $\{SiO_4\}$, dark grey; $\{MoO_6\}$, light grey; O, red. For signal assignment see Table S9.

9.3. ^{27}Al NMR spectra of $[AlMo_6]^{3-}$

Stability of $[AlMo_6]^{3-}$: In ^{27}Al NMR spectra of **$[AlMo_6]^{3-}$** in Hepes pH 7.5. (Figure S31) only one signal at 16.3 ppm is present, which can be attributed to the central Al^{3+} ion in Anderson structure.

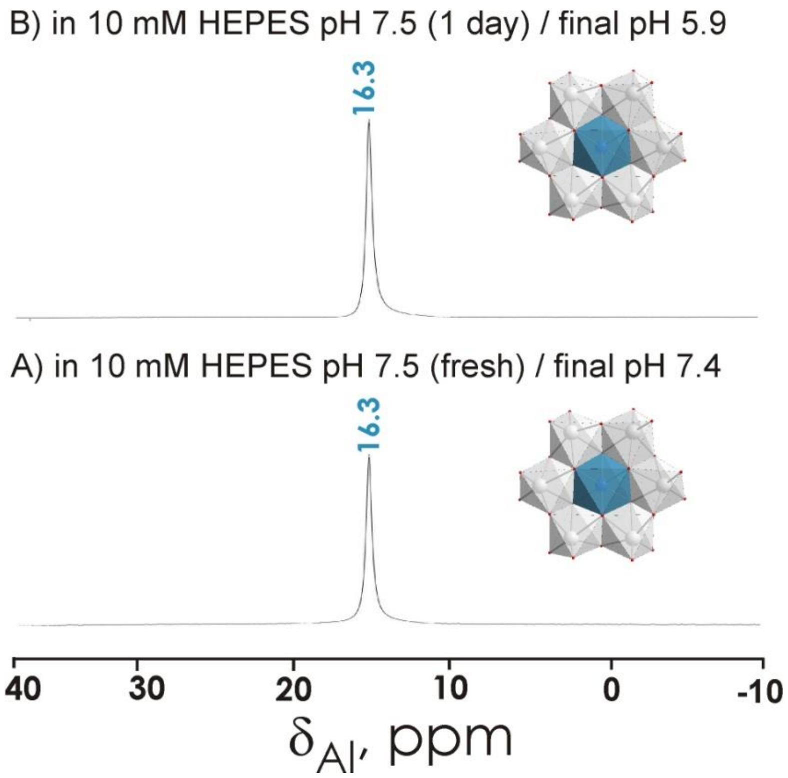

Figure S31. ^{27}Al NMR spectra of 3 mM solutions of **$[AlMo_6]^{3-}$** recorded in 10 mM Hepes pH 7.5 buffer A) immediately after preparation; B) 1 day after preparation. In polyhedral representation of $[Al^{III}(OH)_6Mo^{VI}{}_6O_{18}]^{3-}$ {AlO_6} is shown in blue matching shifts assignment with the same color in A) and B). Color code: {MoO_6}, transparent grey; {AlO_6}, blue; O, red. For signal assignment see Table S9.

9.4. ^{27}Al NMR spectra of [AlW$_{12}$]$^{5-}$

Stability of [AlW$_{12}$]$^{5-}$: In agreement with previous reports **[AlW$_{12}$]$^{5-}$** was shown to be stable at pH 7.5 (Figure S32).

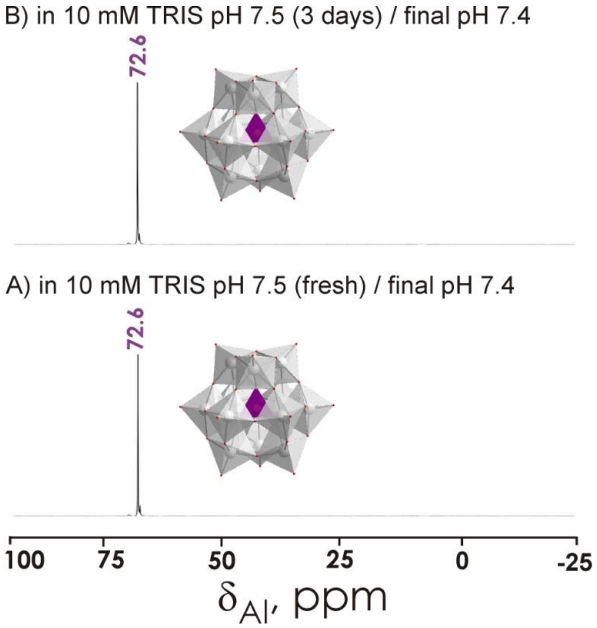

Figure S32. ^{27}Al NMR spectra of 3 mM solutions of **[AlW$_{12}$]$^{5-}$** recorded in 10 mM Tris–HCl pH 7.5 buffer A) immediately after preparation; B) 3 days after preparation. In polyhedral representation of [α-Al^{III}W^{VI}$_{12}$O$_{40}$]$^{5-}$ {AlO$_4$} is shown in purple matching shifts assignment with the same color in A) and B). Color code: {WO$_6$}, transparent grey; {AlO$_4$}, purple; O, red. For signal assignment see Table S9.

9.5. ^{27}Al NMR spectra of [GeAlW$_{11}$]$^{5-}$

Stability of [GeAlW$_{11}$]$^{5-}$: In the solid state, Al-substituted germanotungstate was isolated as protonated anion [α-Al^{III}Ge^{IV}W^{VI}$_{11}$O$_{39}$(H$_2$O)]$^{5-}$. After the dissolution in Tris-HCl buffer at pH 7.5 this anion may be deprotonated to form a five-charged anion. Since the investigation of POM protonation state is a tricky task, only the stability of this anion was tested by ^{27}Al NMR, indicating its integrity at pH 7.5 (Figure S33).

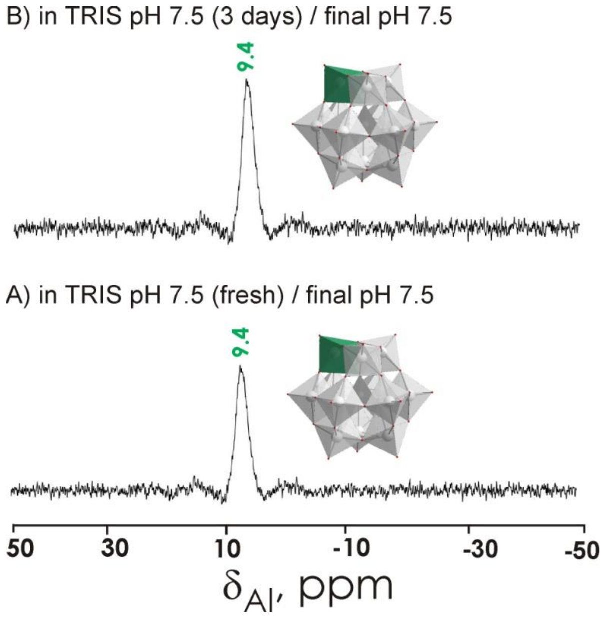

Figure S33. ^{27}Al NMR spectra of 3 mM solutions of **[GeAlW$_{11}$]$^{5-}$** recorded in 10 mM Tris-HCl pH 7.5 buffer A) immediately after preparation; B) 3 days after preparation. In polyhedral representation of [α-Ge^{IV}Al^{III}(H$_2$O)W^{VI}$_{11}$O$_{39}$]$^{5-}$ {AlO$_6$} is shown in green matching shifts assignment with the same color in A) and B). Color code: {WO$_6$}, transparent grey; {GeO$_4$}, grey; {AlO$_6$}, purple; O, red. For signal assignment see Table S9.

9.6. ^{31}P NMR spectra of [(ZrPW$_{11}$)$_2$]$^{8-}$

Stability of [(PZrW$_{11}$)$_2$]$^{8-}$: The sandwich anion [{α-$P^{V}W^{VI}_{11}O_{39}Zr^{IV}$(μ-OH)($H_2O$)}$_2$]$^{8-}$ can be present in solution in fast equilibrium with the monomeric form 1 : 1 Zr(IV)–Keggin POM anion [α-$P^{V}W^{VI}_{11}O_{39}Zr^{IV}$(μ-OH)($H_2O$)]$^{4-}$ that was proved by DOSY and EXSY NMR in combination with DFT calculations.[30,42] Based on this data, **[(PZrW$_{11}$)$_2$]$^{8-}$** was chosen for testing in this research. The ^{31}P NMR spectra of freshly prepared and 1 day aged 10 mM Hepes pH 7.5 solution of **[(PZrW$_{11}$)$_2$]$^{8-}$** remain unchanged with intense signal at –13.7 ppm (Figure S34).

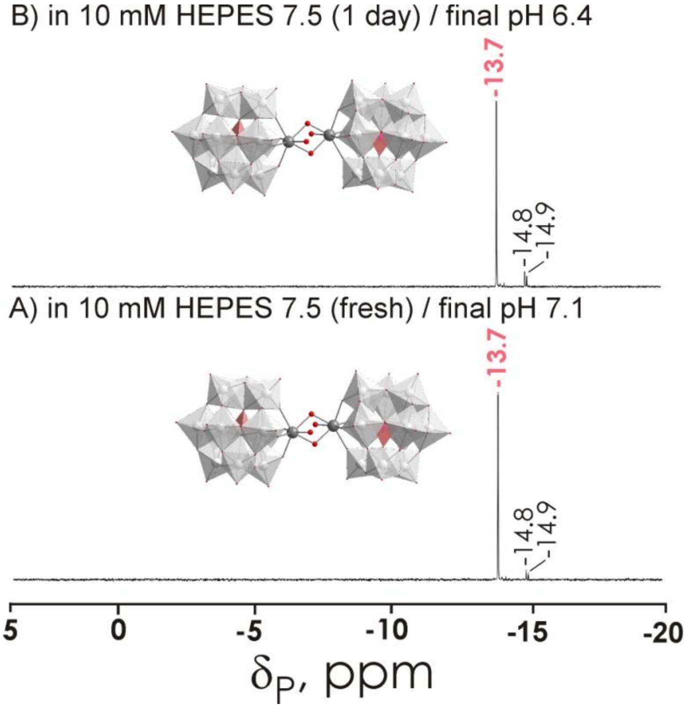

Figure S34. ^{31}P NMR spectra of 3 mM solutions of **[(ZrPW$_{11}$)$_2$]$^{8-}$** recorded in 10 mM Hepes pH 7.5 buffer A) immediately after preparation; B) 1 day after preparation. In polyhedral representation of [{α-$P^{V}W^{VI}_{11}O_{39}Zr^{IV}$(μ-OH)($H_2O$)}$_2$]$^{8-}$ {PO_4} is shown in pink matching shifts assignment with the same color in A) and B). Color code: {WO_6}, transparent grey; {PO_4}, pink; Zr, grey; O, red. For signal assignment see Table S9.

9.7. ^{31}P NMR spectra of $[PW_{12}]^{3-}$

Stability of $[PW_{12}]^{3-}$: According to ^{31}P NMR spectra $\mathbf{[PW_{12}]^{3-}}$ (Figure S36) in 10 mM Mes, 107 mM NaCl, pH 5.5, there is less than 5% of the intact $[\alpha\text{-}P^{V}W^{VI}_{12}O_{40}]^{3-}$ Keggin POT present and the predominant species is monolacunary anion $[\alpha\text{-}P^{V}W^{VI}_{11}O_{39}]^{7-}$. In the stock solution (10 mM acetic acid/sodium acetate pH 4) based on ^{31}P peaks integration the content of $[\alpha\text{-}P^{V}W^{VI}_{12}O_{40}]^{3-}$ is only around 30% (Figure S35).

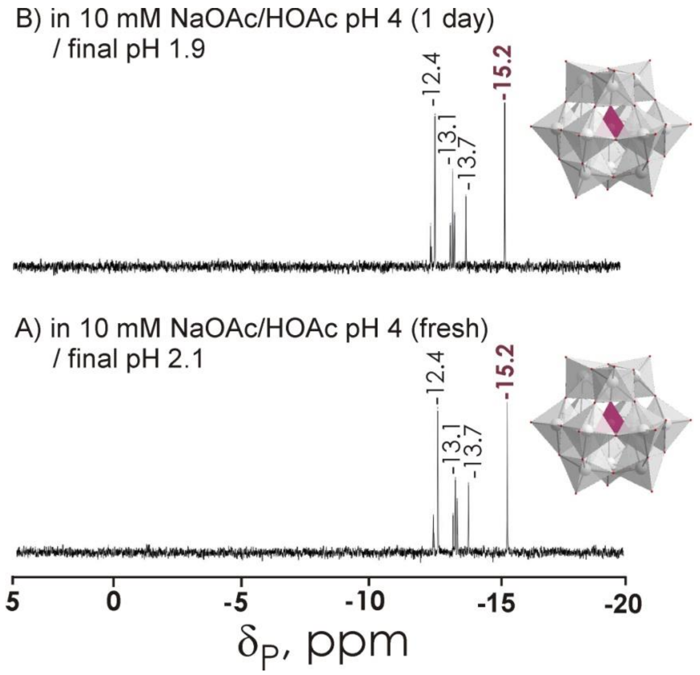

Figure S35. ^{31}P NMR spectra of 3 mM solutions of $\mathbf{[PW_{12}]^{3-}}$ in 10 mM NaOAc/HOAc pH 4 buffer A) immediately after preparation; B) 1 day after preparation. In polyhedral representation of $[\alpha\text{-}P^{V}W^{VI}_{12}O_{40}]^{3-}$ $\{PO_4\}$ is shown in burgundy matching shifts assignment with the same color in A) and B). Color code: $\{WO_6\}$, transparent grey; $\{PO_4\}$, burgundy; O, red. For signal assignment see Table S9.

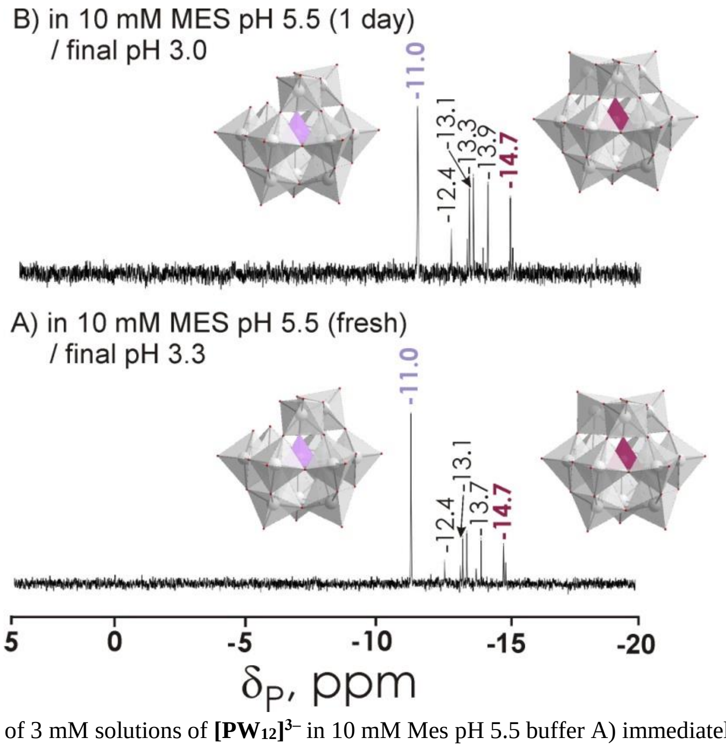

Figure S36. ^{31}P NMR spectra of 3 mM solutions of $\mathbf{[PW_{12}]^{3-}}$ in 10 mM Mes pH 5.5 buffer A) immediately after preparation; B) 1 day after preparation. In polyhedral representation of $[\alpha\text{-}P^{V}W^{VI}_{12}O_{40}]^{3-}$ $\{PO_4\}$ is shown in burgundy and in purple for $[\alpha\text{-}P^{V}W^{VI}_{11}O_{39}]^{7-}$ matching shifts assignment with the same color in A) and B). Color code: $\{WO_6\}$, transparent grey; $\{PO_4\}$, burgundy, purple; O, red. For signal assignment see Table S9.

9.8. ^{183}W NMR spectra of $[SiW_{12}]^{4-}$

Stability of $[SiW_{12}]^{4-}$: Based on ^{183}W NMR (Figure S37) in the solution of **$[SiW_{12}]^{4-}$** in Hepes buffer pH 7.5 two anions are present: intact $[\alpha\text{-}Si^{IV}W^{VI}_{12}O_{40}]^{4-}$ and monolacunary $[\alpha\text{-}Si^{IV}W^{VI}_{11}O_{39}]^{8-}$.

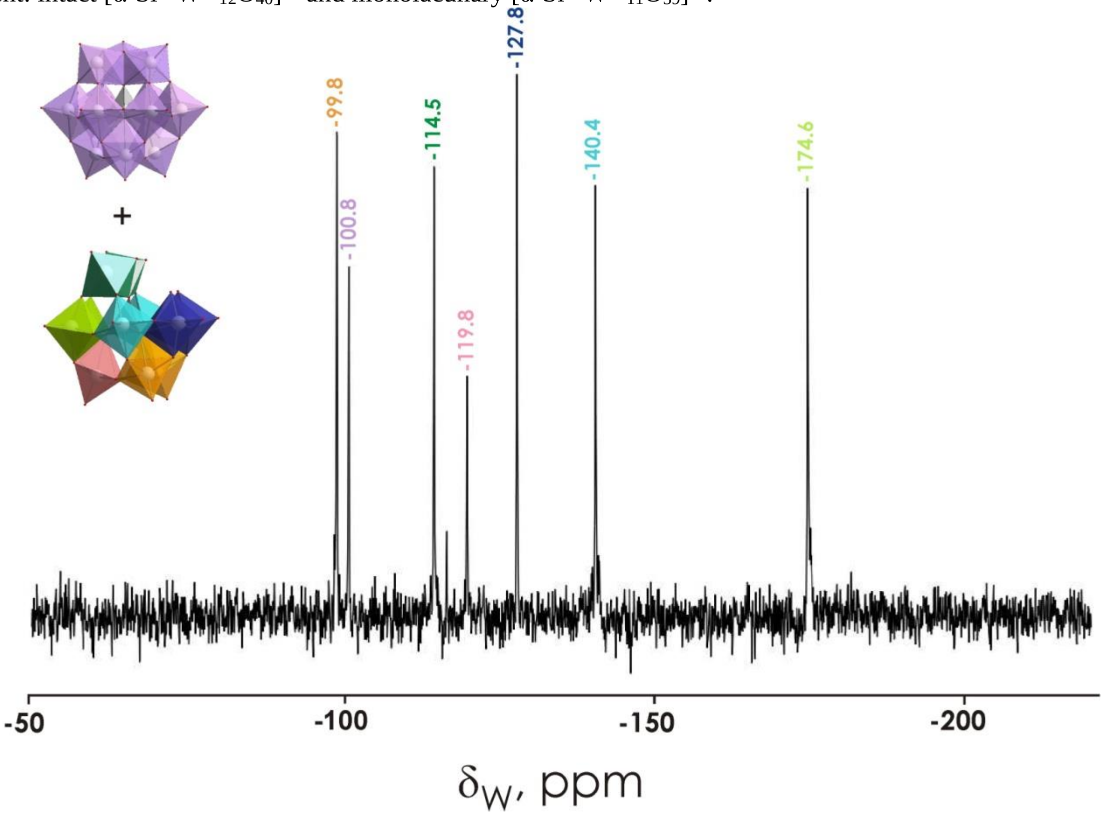

Figure S37. ^{183}W NMR spectra of 10 mM solutions of **$[SiW_{12}]^{4-}$** recorded in 33 mM Hepes pH 7.5 buffer (final pH is 6.8). In polyhedral representation of $[Si^{IV}W^{VI}_{12}O_{40}]^{4-}$ one type $\{WO_6\}$ is shown in purple and six types of $\{WO_6\}$ in mono-lacunary $[Si^{IV}W^{VI}_{11}O_{39}]^{8-}$ are shown in six different colors matching shifts assignment with the same color. Color code: $\{WO_6\}$, green, lime, indigo, blue, pink, and orange; $\{SiO_4\}$, dark grey; $\{MoO_6\}$, light grey; O, red. For signal assignment see Table S9.

10. ESI-MS

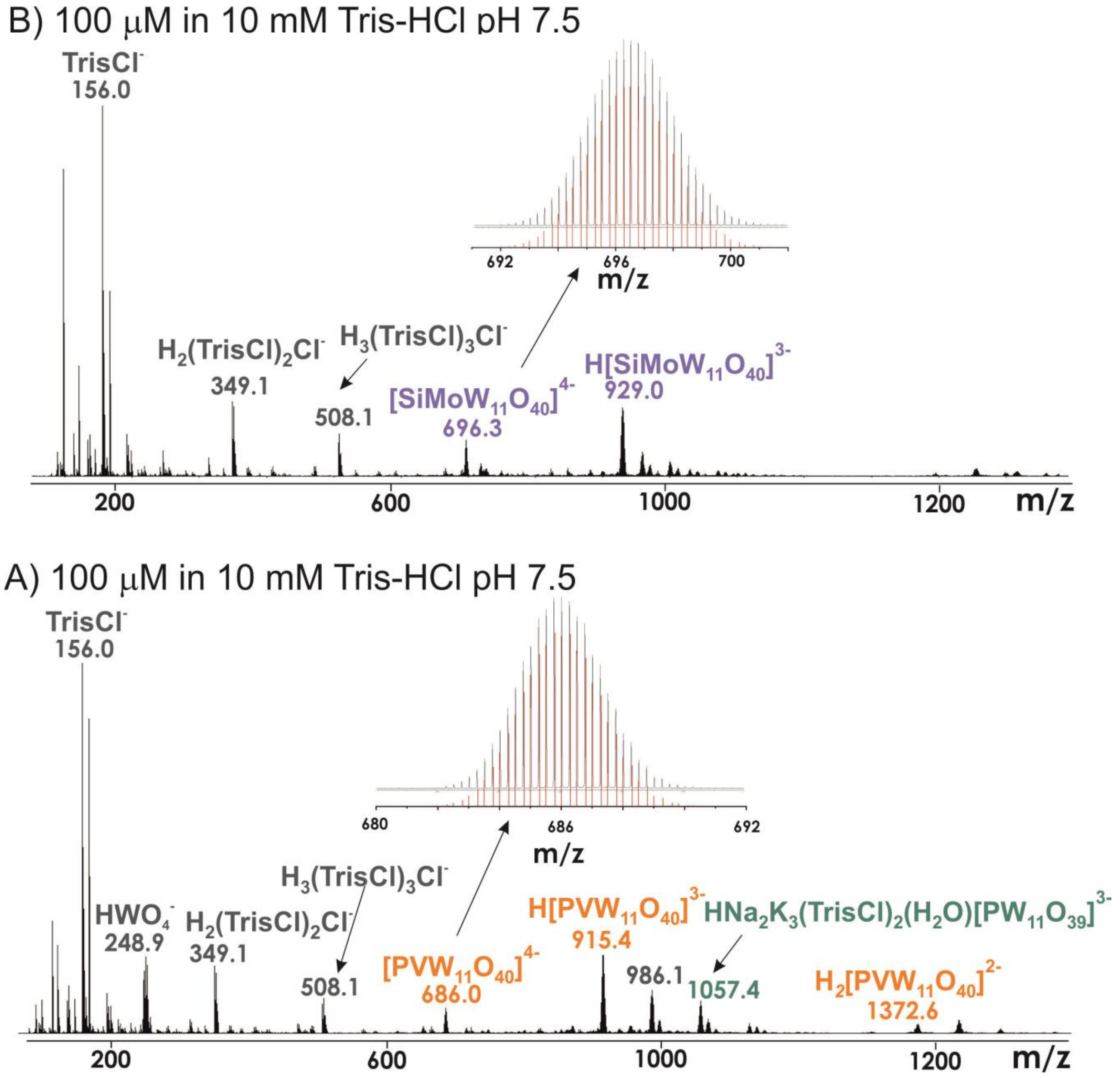

Figure S38. ESI mass spectrum of 100 μM **$[PVW_{11}]^{4-}$** (A) and **$[SiMoW_{11}]^{4-}$** (B) in 10 mM Tris-HCl buffer pH 7.5. The spectra were recorded in negative ion mode within the *m*/*z* range of 100 to 1300, and the spectrometer was calibrated using the standard tune-mix to ensure an accuracy of approximately 5 ppm in the *m*/*z* region of 100−1500. In the case of the quadrupole anions, the overlap between the experimental (black) and simulated (red) envelopes is depicted in the insert. Following the initial injection of a Tris-HCl solution, only the signals of Tris-HCl complexes were visible. Subsequent flushing with H_2O allowed us to distinguish the POM envelopes.

11. References

[1] M. Cholewa, G. J. F. Legge, H. Weigold, G. Holan, C. J. Birch, *Life Sci.* **1994**, *54*, 1607.
[2] L. Ni, P. Greenspan, R. Gutman, C. Kelloes, M. A. Farmer, F. D. Boudinot, *Antiviral Res.* **1996**, *32*, 141.
[3] X. Wang, F. Li, S. Liu, M. T. Pope, *J. Inorg. Biochem.* **2005**, *99*, 452.
[4] G. Modugno, E. Fabbretti, A. Dalle Vedove, T. Da Ros, C. Maccato, H. S. Hosseini, M. Bonchio, M. Carraro, *Eur. J. Inorg. Chem.* **2018**, *2018*, 4955.
[5] H. Nabika, Y. Inomata, E. Itoh, K. Unoura, *RSC Adv.* **2013**, *3*, 21271.
[6] H. Nabika, A. Sakamoto, T. Motegi, R. Tero, D. Yamaguchi, K. Unoura, *J. Phys. Chem. C* **2016**, *120*, 15640.
[7] A. Sakamoto, K. Unoura, H. Nabika, *J. Phys. Chem. C* **2018**, *122*, 1404.
[8] N. Fukuda, T. Yamase, Y. Tajima, *Biol. Pharm. Bull.* **1999**, *22*, 463.
[9] Y. Tajima, *Biomed. Res.* **2002**, *23*, 115.
[10] G. Zhang, B. Keita, C. T. Craescu, S. Miron, P. de Oliveira, L. Nadjo, *J. Phys. Chem. B* **2007**, *111*, 11253.
[11] G. Zhang, B. Keita, C. T. Craescu, S. Miron, P. de Oliveira, L. Nadjo, *Biomacromolecules* **2008**, *9*, 812.
[12] L. Zheng, Y. Ma, G. Zhang, J. Yao, B. S. Bassil, U. Kortz, B. Keita, P. de Oliveira, L. Nadjo, C. T. Craescu, S. Miron, *Eur. J. Inorg. Chem.* **2009**, *2009*, 5189.
[13] L. Zheng, Y. Ma, G. Zhang, J. Yao, B. Keita, L. Nadjo, *Phys. Chem. Chem. Phys.* **2010**, *12*, 1299.
[14] Y. Zhou, L. Zheng, F. Han, G. Zhang, Y. Ma, J. Yao, B. Keita, P. de Oliveira, L. Nadjo, *Colloids Surf. A Physicochem. Eng. Asp.* **2011**, *375*, 97.
[15] K. Stroobants, D. Saadallah, G. Bruylants, T. N. Parac-Vogt, *Phys. Chem. Chem. Phys.* **2014**, *16*, 21778.
[16] K. Stroobants, V. Goovaerts, G. Absillis, G. Bruylants, E. Moelants, P. Proost, T. N. Parac-Vogt, *Chem. Eur. J.* **2014**, *20*, 9567.
[17] T. Zhang, H.-W. Li, Y. Wu, Y. Wang, L. Wu, *Chem. Eur. J.* **2015**, *21*, 9028.
[18] T. Zhang, D.-Y. Fu, Y. Wu, Y. Wang, L. Wu, *Dalton Trans.* **2016**, *45*, 15457.
[19] Y. Liu, X. Yuan, W. Wang, Y. Wu, L. Wu, *New J. Chem.* **2018**, *42*, 17339.
[20] S. Fabbian, G. Giachin, M. Bellanda, C. Borgo, M. Ruzzene, G. Spuri, A. Campofelice, L. Veneziano, M. Bonchio, M. Carraro, R. Battistutta, *Front. Mol. Biosci.* **2022**, *9*, 906390.
[21] M. Nishihara, F. Perret, T. Takeuchi, S. Futaki, A. N. Lazar, A. W. Coleman, N. Sakai, S. Matile, *Org. Biomol. Chem.* **2005**, *3*, 1659.
[22] M. M. Mady, M. M. Ghannam, *Afr. J. Pharm. Pharmacol.* **2011**, *5,* 898.
[23] S. K. Lim, C. Sandén, R. Selegård, B. Liedberg, D. Aili, *Sci. Rep.* **2016**, *6*, 21123.
[24] For the different anionic formulations previously reported in the literature, we found that the combination DMPE/DPPG/CHOL, in a molar ratio of 1/2/1, is the most stable under the experimental conditions; vesicles retained stability and ensured reproducible measurements for 1 month.
[25] A. Hennig, H. Bakirci, W. M. Nau, *Nat. Methods* **2007**, *4*, 629.
[26] D. M. Bailey, A. Hennig, V. D. Uzunova, W. M. Nau, *Chem. Eur. J.* **2008**, *14*, 6069.
[27] W. M. Nau, G. Ghale, A. Hennig, H. Bakirci, D. M. Bailey, *J. Am. Chem. Soc.* **2009**, *131*, 11558.
[28] M. Florea, W. M. Nau, *Org. Biomol. Chem.* **2010**, *8*, 1033.
[29] G. Ghale, V. Ramalingam, A. R. Urbach, W. M. Nau, *J. Am. Chem. Soc.* **2011**, *133*, 7528.
[30] H. G. T. Ly, G. Absillis, T. N. Parac-Vogt, *Dalton Trans.* **2013**, *42*, 10929.
[31] H. G. T. Ly, G. Absillis, T. N. Parac-Vogt, *New J. Chem.* **2016**, *40*, 976.
[32] E. Vandermarliere, M. Mueller, L. Martens, *Mass Spectrom. Rev.* **2013**, *32*, 453.
[33] X. L. Guo, L. Li, D. Q. Wei, Y. S. Zhu, K. C. Chou, *Amino Acids* **2008**, *35*, 375.
[34] R. Thouvenot, M. Fournier, R. Franck, C. Rocchiccioli-Deltcheff, *Inorg. Chem.* **1984**, *23*, 598.
[35] S. Yao, C. Falaise, A. A. Ivanov, N. Leclerc, M. Hohenschutz, M. Haouas, D. Landy, M. A. Shestopalov, P. Bauduin, E. Cadot, *Inorg. Chem. Front.* **2021**, *8*, 12.
[36] K. Nomiya, Y. Saku, S. Yamada, W. Takahashi, H. Sekiya, A. Shinohara, M. Ishimaru, Y. Sakai, *Dalton Trans.* **2009**, 5504.
[37] G. H. A. Téazéa, R. G. Finke, D. K. Lyon in *Inorg. Synth., Vol. 27* (Ed.: A. P. Ginsberg), John Wiley & Sons,Inc., **1990**, pp. 85.
[38] I. A. Weinstock, J. J. Cowan, E. M. G. Barbuzzi, H. Zeng, C. L. Hill, *J. Am. Chem. Soc.* **1999**, *121*, 4608.
[39] E. Tanuhadi, N. I. Gumerova, A. Prado-Roller, M. Galanski, H. Čipčić-Paljetak, D. Verbanac, A. Rompel, *Inorg. Chem.* **2021**, *60*, 28.
[40] S. Manikumari, V. Shivaiah, S. K. Das, *Inorg. Chem.* **2002**, *41*, 6953.
[41] R. I. Maksimovskaya, G. M. Maksimov, *Coord. Chem. Rev.* **2019**, *385*, 81.
[42] O. A. Kholdeeva, G. M. Maksimov, R. I. Maksimovskaya, M. P. Vanina, T. A. Trubitsina, D. Y. Naumov, B. A. Kolesov, N. S. Antonova, J. J. Carbó, J. M. Poblet, *Inorg. Chem.* **2006**, *45*, 7224.
